# Reprogrammed Fibrotic Niche Fuels Lung Cancer Initiation and Reciprocal Remodeling

**DOI:** 10.7150/ijbs.127307

**Published:** 2026-01-22

**Authors:** Zhufeng Hu, Wu Dan, Mengran Xi, Zhengyuan Fang, Kunlun Feng, Jie Mei, Zhang Ting, Baojun Liu, Zhiwen Luo

**Affiliations:** 1 Department of Integrative Medicine, Huashan Hospital Affiliated to Fudan University, Shanghai 200040, China.; 2 City University of Hong Kong, China.; 3 Department of Gastrointestinal Surgery, Renji Hospital, Shanghai 200127, China.; 4 First Clinical College, Shandong University of Traditional Chinese Medicine, China.; 5 The First Clinical Medicine College, Nanjing Medical University, Nanjing, 211166, China.; 6 Department of Sports Medicine, Huashan Hospital Affiliated to Fudan University, Shanghai 200040, China.; 7 Fudan Zhangjiang Institute, Fudan University, Shanghai, China.; 8 Fudan University - Dr. Kong Joint Research Center for Sports Medicine and Health Footwear, Fudan University Institute of Sports Medicine (Jinqiao Laboratory), Shanghai, China.; 9 Department of Orthopaedics, Jiaxing Key Laboratory of Basic Research and Clinical Translation on Orthopedic Biomaterials, The Second Affiliated Hospital of Jiaxing University (Sports Hospital of Jiaxing), 1518 North Huancheng Road, Jiaxing 314000, China.

**Keywords:** pulmonary fibrotic microenvironment, lung cancer, cancer-associated fibroblasts, mechanotransduction, metabolic reprogramming, microbiome dysbiosis

## Abstract

Pulmonary Fibrosis (PF), an end-stage manifestation of interstitial lung diseases, is associated with largely unfavorable prognoses. Lung cancer (LC), a leading cause of nationally cancer-related mortality with progressively increasing incidence, exhibits pathological interconnections with PF. The chronic remodeling of the pulmonary microenvironment—including cellular components, extracellular matrix (ECM), inflammatory cytokine networks, and metabolic reprogramming—represents the core pathogenic mechanism underlying PF-LC comorbidity. This review systematically elaborates how the fibrotic microenvironment promotes malignant transformation of lung cancer via chronic inflammation, increased matrix stiffness, immunosuppressive regulation, and epigenetic modulation. Furthermore, we investigate the bidirectional crosstalk by which LC progression reciprocally modulates fibrotic processes. Finally, we integrate current clinical challenges and propose novel therapeutic strategies targeting the fibrotic microenvironment to address this lethal pathophysiological synergy.

## 1. Introduction

PF, the end-stage manifestation of interstitial lung diseases, is characterized by aberrant activation and proliferation of fibroblasts accompanied by massive ECM deposition, leading to destructive remodeling of lung architecture. Notably, the PF microenvironment shares striking similarities with the tumor microenvironment (TME), particularly in ECM remodeling, chronic inflammation, and immunosuppression^1^. For instance, activated myofibroblasts in PF and cancer-associated fibroblasts (CAFs) in TME both drive pathological progression via secretion of transforming growth factor (TGF)-β, interleukin-6 (IL-6), and other profibrotic cytokines^2^-^5^. However, the PF microenvironment exhibits unique protumorigenic features: compared to the dynamic ECM remodeling in TME, PF displays significantly higher and irreversible ECM crosslinking, resulting in sustained elevation of matrix stiffness. This biomechanical stress activates the yes-associated protein (YAP)/transcriptional coactivator with PDZ-binding motif (TAZ) mechanical signaling pathway, driving alveolar epithelial cells (AECs) toward epithelial-mesenchymal transition (EMT)—a process associated with elevated genomic instability compared to normal lung tissue^6^-^10^.

Idiopathic pulmonary fibrosis (IPF), the most prevalent and progressive PF subtype with unknown etiology, high symptom burden, and poor prognosis, is marked by persistent TGF-β signaling abnormalities in its microenvironment. Beyond promoting fibrosis, TGF-β signaling via Smad3-dependent pathways induces infiltration of immunosuppressive cells (e.g., regulatory T cells and M2 macrophages)^11^-^13^. While this immunosuppressive landscape shares functional parallels with Programmed cell death-1 (PD-1)/Programmed death-ligand 1 (PD-L1)-mediated T cell exhaustion in TME, PF-associated myeloid cells (e.g., macrophages) predominantly drive fibrotic rather than oncogenic processes^14^,^15^. Non-IPF PF variants, often arising from autoimmune diseases or environmental exposures, frequently progress to progressive pulmonary fibrosis (PPF) in 30-50% of cases, exhibiting comparable disease burdens and outcomes to IPF^16^. A critical distinction lies in the hypoxic microenvironment of PF lungs, where hypoxia-inducible factor (HIF)-1α upregulation elevates PD-L1 expression in epithelial cells, causing CD8+ T cell exhaustion—a mechanism analogous to hypoxia-driven immune evasion in TME. However, PF-associated hypoxic zones are more extensive and persistent, potentially accelerating multifocal lung carcinogenesis^17^-^20^. These pathobiological overlaps and divergences underscore the need to dissect PF-TME crosstalk for developing comorbidity-specific therapeutics.

Lung cancer remains the leading cause of cancer-related mortality globally, with incidence rates steadily rising^21^. Epidemiological evidence strongly implicates PF, particularly IPF, as a significant risk factor for lung carcinogenesis, showing a 3.5- to 7.3-fold increased lung cancer risk even after adjusting for smoking exposure, a disparity most pronounced in Asian populations^22^-^27^. This robust association may stem from synergistic microenvironmental evolution: while the PF microenvironment provides a pro-cancerous "soil" (e.g., elevated matrix stiffness, inflammatory cytokines), lung cancer cells reciprocally recruit fibroblasts via connective tissue growth factor (CTGF) and lysyl oxidase like 2 (LOXL2) secretion, forming a feedforward "fibrosis-tumor" loop distinct from the unidirectional stromal regulation in TMEs^1^,^28^-^31^. Mechanistic studies reveal that chronic inflammation in PF microenvironments generates high reactive oxygen species (ROS) levels, inducing TP53 mutations in AECs and directly promoting carcinogenesis^32^,^33^. Notably, patients with concurrent lung cancer and PF exhibit 1.51-fold higher all-cause mortality compared to IPF patients without malignancy^27^, potentially attributed to fibroblast activation via miR-21-mediated crosstalk between CAFs and myofibroblasts, which synergistically drives ECM stiffening and chemoresistance—a phenomenon rarely observed in TMEs of isolated lung cancer^34^,^35^. Therapeutic targeting of this axis shows promise: antifibrotic treatment (e.g., nintedanib) reduces all-cause mortality by 39% in IPF patients with comorbid lung cancer^27^, while its fibroblast growth factor receptor (FGFR) signaling inhibition confers dual anti-fibrotic and anticancer effects^36^. These findings underscore the potential of targeting PF-TME convergent pathways for synergistic therapeutic benefits over conventional oncology approaches.

Despite these advances, critical knowledge gaps persist: 1. How do fibrotic microenvironments uniquely drive lung cancer initiation and early evolution through chronic inflammation, genomic instability, epigenetic reprogramming, biomechanical signaling, immunosuppression, and stem cell niches? 2. How do tumor-derived factors (e.g., pro-fibrotic cytokines, metabolic reprogramming) remodel fibrotic microenvironments during cancer progression, exacerbating therapeutic resistance and poor outcomes through bidirectional feedback loops? This review systematically dissects PF-microenvironment (PFME)-TME interactions, emphasizing PFME-driven carcinogenic mechanisms and TME-mediated fibrotic reinforcement. We further identify actionable targets at the intersection of these pathologies for novel therapeutic strategies.

## 2. Key Features of the PFME​

**Figure 1:**
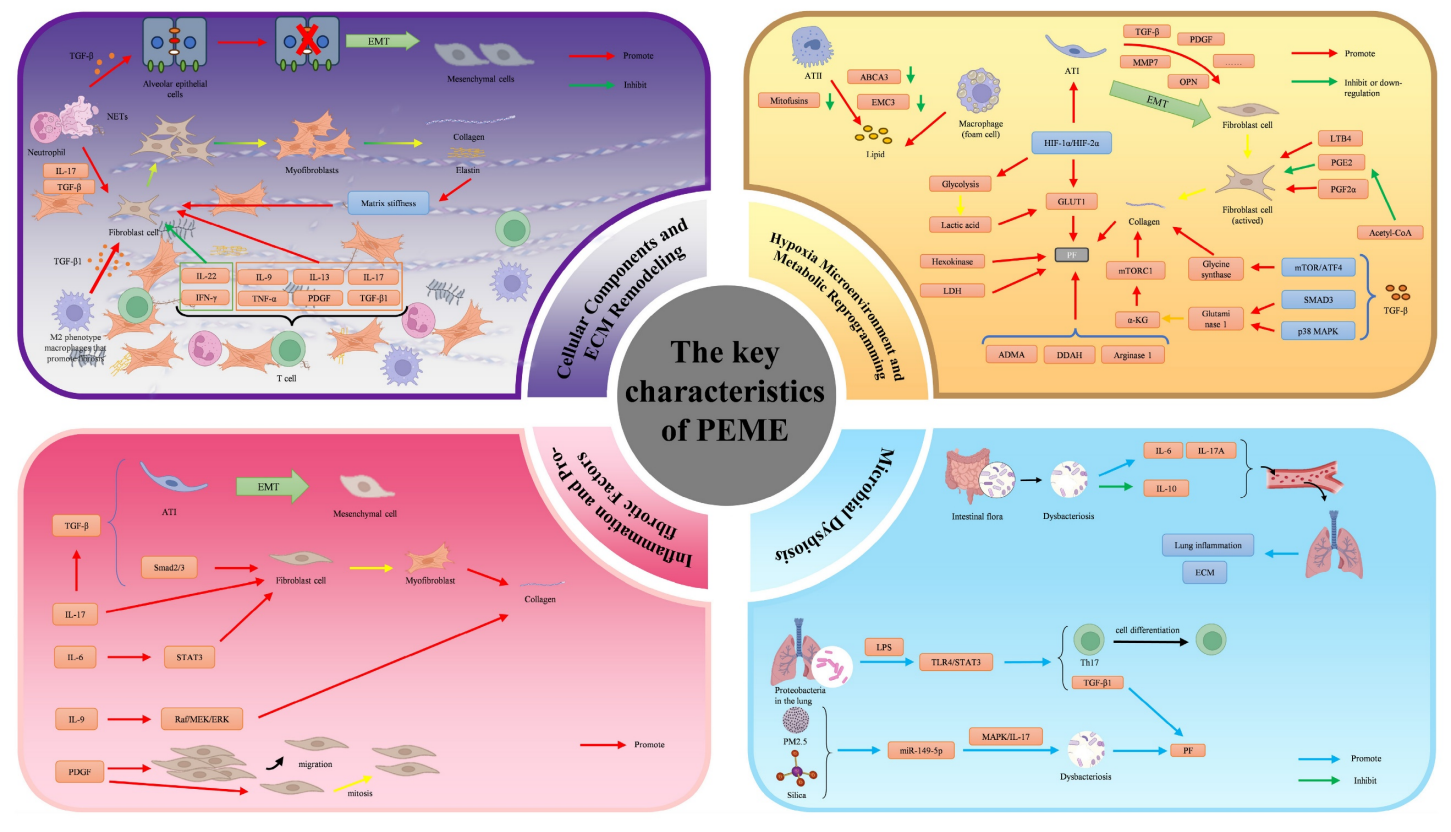
** Schematic diagram of the key characteristic of the PFME.** The key characteristics of the pulmonary fibrotic microenvironment encompass cellular components, ECM remodeling, inflammation and pro-fibrotic factor networks, hypoxic microenvironment, metabolic reprogramming, and microbiome dysbiosis. Cellular components of the pulmonary fibrotic microenvironment primarily consist of fibroblasts, inflammatory cells, and epithelial cells. Fibroblasts differentiate into myofibroblasts. Excessive myofibroblasts remain persistently activated in fibrotic lungs and secrete ECM components such as collagen type I and α-SMA. Inflammatory cells generate various inflammatory cytokines and pro-fibrotic factors, thereby driving fibrosis. Alveolar epithelial cells undergo EMT, which promotes the development of PF. The ECM is remodeled by various cells. Increased matrix stiffness further promotes the progression of PF. Hypoxia induces alveolar epithelial cells to secrete pro-fibrotic factors and can affect fibrosis through metabolic alterations. Metabolism in PF is reprogrammed, which further promotes PF. Inflammatory cytokines and pro-fibrotic factors synergistically drive disease progression through complex interactive networks. In PF, structural and functional dysbiosis of the gut and lung microbiota exacerbates the inflammatory and fibrotic processes via multidimensional mechanisms. Copyrighted from the BioRender platform.

The key characteristics of the PFME encompass cellular components, ECM remodeling, inflammatory networks, hypoxia, metabolic reprogramming, and microbiome dysbiosis. These features interact synergistically to drive fibrotic progression while creating a "soil" for lung cancer initiation and early evolution through mechanisms such as chronic inflammation, genomic instability, mechanical signaling, and immunosuppression. Below is a brief overview of each characteristic, with emphasis on their direct associations with lung carcinogenesis. (Fig. 1).

### 2.1 ​​Cellular Components​

The cellular components within the PFME, including fibroblasts, inflammatory cells, and epithelial cells, drive fibrosis through abnormal activation and intercellular crosstalk, while directly contributing to lung cancer initiation.

#### 2.1.1 ​​Activated Fibroblasts/Myofibroblasts​

Activated myofibroblasts are the core effector cells of PF, promoting fibrosis by secreting ECM components (such as collagen I) and expressing the α-SMA contractile apparatus^37^. In the PFME, fibroblasts secrete TGF-β to activate CAFs, thereby establishing a pro-tumor microenvironment^5^. CAF subtypes exhibit heterogeneity. Different CAF subtypes promote malignant cell proliferation, metabolism, stemness, chemotherapy resistance, EMT, invasion, and migration through paracrine signaling, ECM remodeling, and/or metabolite exchange^38^. Myofibroblast-like CAFs (myCAFs) differentiate upon TGF-β induction, highly express α-SMA and fibroblast activating protein (FAP), and dominate excessive ECM production, forming a physical barrier that promotes treatment resistance, but may suppress early tumors via collagen cross-linking; Inflammatory CAFs (iCAFs) rely on IL-1 signaling to secrete inflammatory factors such as IL-6 and CXCL-1, recruit myeloid-derived suppressor cells (MDSCs) and regulatory T cells (Tregs) to suppress immunity, and exhibit dynamic transformation with myCAFs; Antigen-presenting CAFs (apCAFs) present antigens via MHC class II molecules, which can either activate Tregs to enhance immunosuppression or trigger CD4+ T cell anti-tumor responses in specific cancer types; Tumor-suppressive CAFs (rCAFs) secrete Meflin and Asporin to inhibit ECM cross-linking and the TGF-β pathway, suppressing tumor growth in the early stage but decreasing with disease progression^39^. For example, CAFs induce resistance to almonertinib in patients with non-small cell lung cancer (NSCLC) by activating the YAP/TAZ signaling pathway^40^.

#### 2.1.2 Inflammatory Cells

**Figure 2:**
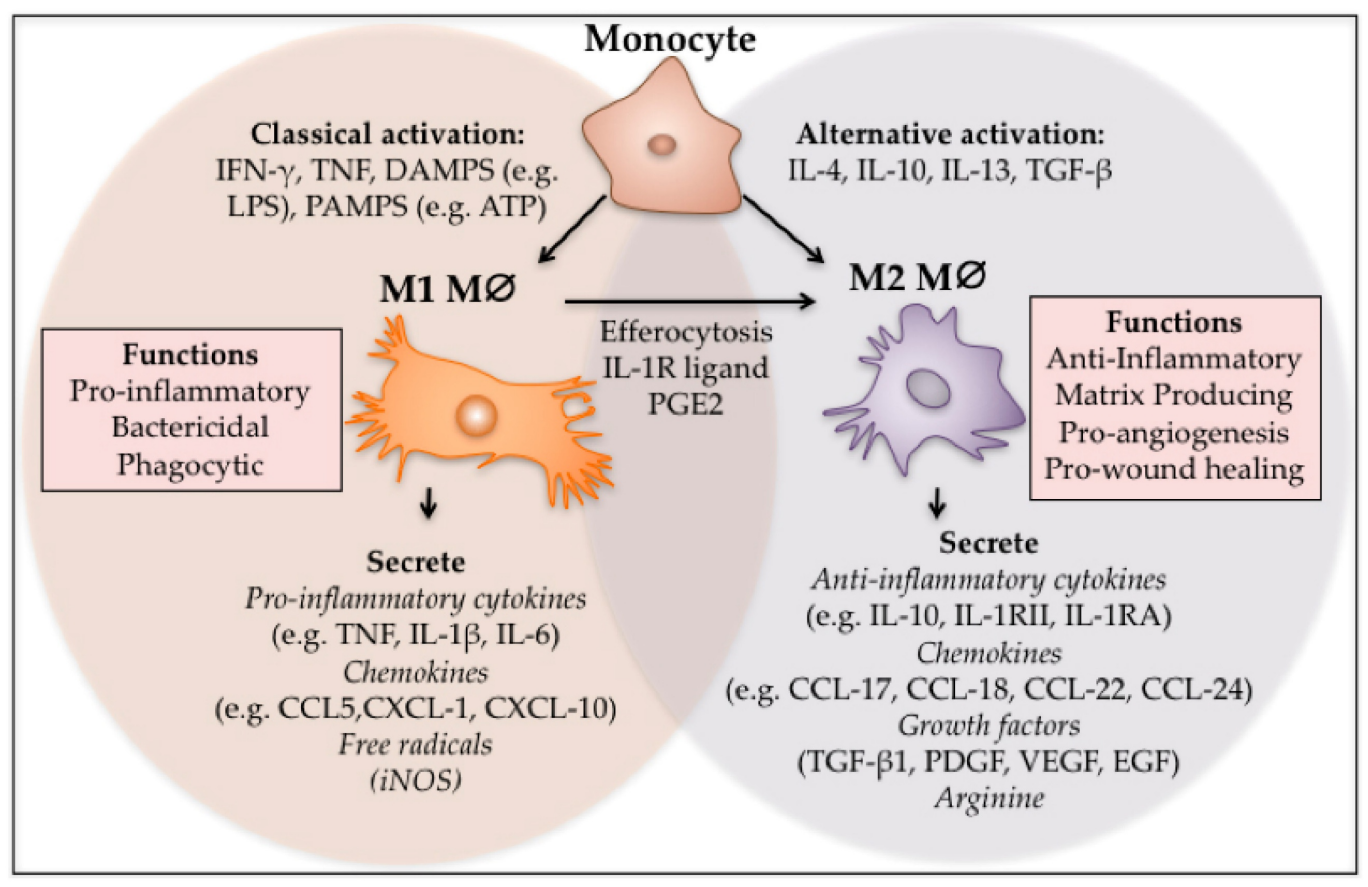
** M1 and M2 polarisation of macrophages.** Monocytes can be classically or alternatively activated to form M1 and M2 macrophages respectively. M1 macrophages can also differentiate into M2 macrophages through local cues and after efferocytosis. The M1 phenotype is pro-inflammatory, phagocytic and bactericidal, while the M2 macrophages act to switch off inflammation and regulate re-vascularisation and wound closure. (Note. From Hesketh et al.^41^ (2017) under the terms of the Creative Commons Attribution International License (CC BY 4.0).).

Inflammatory cells promote malignant transformation in PF through chronic inflammatory signals. Macrophages polarize into M2 phenotypes (such as M2a, M2c, M2d), secrete high levels of TGF-β1, induce fibroblast activation and suppress immune responses, creating a microenvironment permissive for lung cancer growth^41^. Additionally, macrophages secrete cytokines such as tumor necrosis factor-α (TNF-α), IL-1β, cyclooxygenase-2 (COX-2), vascular endothelial growth factor (VEGF), etc., mediating immunosuppression, immune evasion, and metastasis^42^ (Fig. 2).

**Figure 3:**
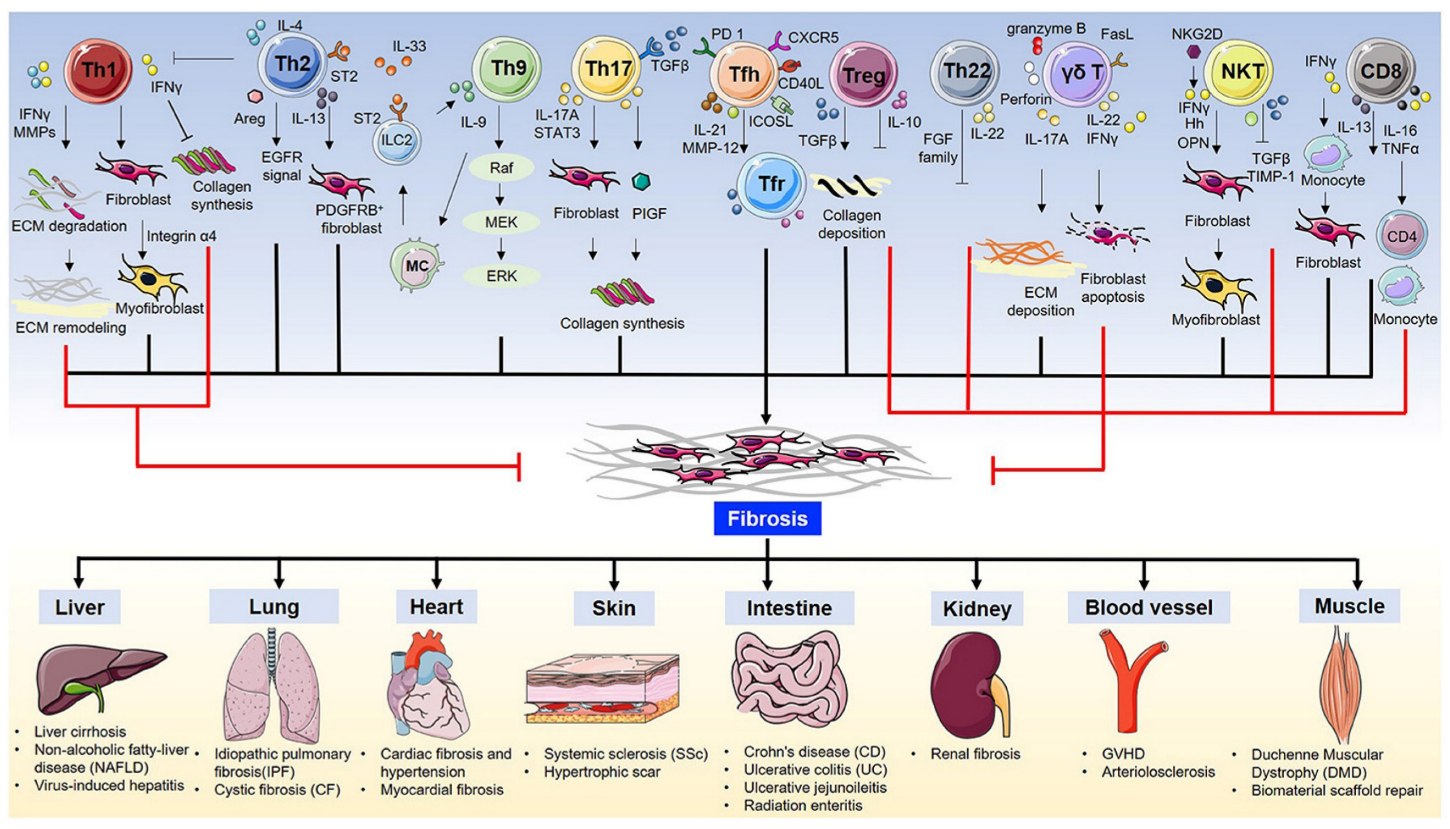
** T cells in fibrosis and fibrotic diseases.** (Note. From Zhang et al.^43^ (2020) under the terms of the Creative Commons Attribution International License (CC BY 4.0).).

T cells play a key role in regulating fibrosis through diverse subtypes and functional polarization. T cell subsets (such as Th2, Th17) drive fibrosis by secreting factors such as IL-13 and IL-17, while suppressing anti-tumor immunity^43^ (Fig. 3). For example, CD4+ T cells can upregulate PD-1 and secrete IL-17A and TGF-β1 via the signal transducer and activator of transcription 3 (STAT3) pathway, promoting PF^44^.

Neutrophils bridge the progression of fibrosis and lung cancer by releasing toxic mediators and forming neutrophil extracellular traps (NETs). By forming neutrophil extracellular traps, neutrophils directly activate fibroblasts and induce epithelial damage and EMT, and generate sustained chronic inflammation mediated by TGF-β and IL-17 signaling, whose components (such as MPO) exacerbate oxidative stress, increase the risk of DNA mutations in lung epithelial cells, thereby directly driving lung cancer initiation^45^.

#### 2.1.3 Epithelial Cells

Repeated injury and alterations in alveolar epithelial cells (AECs), including proliferation and hyperplasia, have been shown to weaken epithelial-mesenchymal crosstalk, leading to aberrant activation of AECs and epithelial cell senescence—all closely associated with pulmonary fibrosis (PF) progression^46^-^48^. Existing evidence indicates that PF is caused by repeated injury to AECs, triggering aberrant interactions between epithelial cells and fibroblasts 49,50. Apoptosis of Type II alveolar epithelial cells (ATII) and inactivation of the Hippo pathway enhance YAP/TAZ signaling, promoting aberrant regeneration 10. This is not only the core mechanism of PF but also provides a basis for the formation of the lung cancer stem cell niche, directly linked to early tumorigenesis. This will be discussed in detail in subsequent sections.

### 2.2 ECM Remodeling

Pathological epithelial remodeling and ECM accumulation are the core pathological features of PF. The ECM is primarily composed of collagen, elastin, glycoproteins, and proteoglycans, and its remodeling process is closely related to fibroblasts^51^. TGF-β signaling drives fibroblast proliferation and differentiation into myofibroblasts, which secrete collagen excessively^41^. As mentioned earlier, macrophages, T cells, and epithelial cells jointly promote ECM crosslinking and deposition via paracrine signals (such as IL-6 and TGF-β). The mechanotransduction pathways that play a key role in PF progression include Rho-associated protein kinase (ROCK), myocardin-related transcription factor (MRTF), YAP, and TAZ^7^,^52^,^53^. Increased matrix stiffness activates the YAP/TAZ pathway via integrins, inducing fibroblast activation and fibrosis^7^. This process forms a positive feedback loop: stiffness enhancement → YAP/TAZ activation → collagen secretion → further hardening. Increased ECM stiffness can hinder the penetration of drugs and immune cells and provide a niche for tumor progression^54^. This suggests the dual role of ECM stiffness in tumor-fibrosis crosstalk.

### 2.3 Inflammation and Pro-fibrotic Factor Network​

In the pathological progression of PF, inflammatory cytokines and pro-fibrotic factors synergistically drive disease advancement through intricate interaction networks. TGF-β exposure during lung tissue injury induces EMT^55^. Additionally, TGF-β promotes fibroblast-to-myofibroblast differentiation via the Smad2/3 pathway, enhancing collagen and fibronectin deposition^56^. IL-6 accelerates fibroblast proliferation by activating the signal transducer and activator of transcription 3 (STAT3) signaling cascade^57^. IL-9 stimulates collagen deposition through the Raf/MEK/ERK pathway^58^. IL-17 directly activates fibroblasts and myofibroblasts, inhibits autophagy, and induces EMT in epithelial cells by promoting TGF-β production^59^. The PDGF family induces fibroblast migration while exerting anti-apoptotic and mitogenic effects on ECM-producing fibroblasts and myofibroblasts, thereby accelerating fibrotic disease progression^60^. These pro-fibrotic factors activate fibroblasts or enhance collagen deposition via distinct signaling pathways to advance PF. Conversely, fibroblasts or ECM remodeling exert feedback regulation on this pro-fibrotic factor network (Fig. 1).

### 2.4 Hypoxic Microenvironment​

Hypoxia-induced lung tissue injury activates hypoxia-responsive pathways, with core regulators HIF-1α/HIF-2α playing pivotal roles in both IPF and lung cancer. Hypoxia induces alveolar epithelial cells to secrete pro-fibrotic factors such as TGF-β and PDGF, activating fibroblasts through enhanced glycolysis, upregulation of glucose transporter 1 (GLUT1), ECM deposition, mitochondrial ROS/tricarboxylic acid (TCA) metabolite-mediated macrophage activation, and induction of alveolar epithelial apoptosis or EMT, thereby driving IPF fibrosis^61^,^62^. Simultaneously, hypoxia upregulates matrix metalloproteinases 7 (MMP7)/MMP14 to promote lung cancer invasion/metastasis, activates Warburg metabolism for tumor survival, and suppresses immunity via lactate and other metabolites^62^. IPF and lung cancer share HIFs-MMPs pathways and metabolic mechanisms, suggesting targeted therapies could simultaneously intervene in fibrosis and tumor progression.

### 2.5 Metabolic Reprogramming​

In PF, metabolic reprogramming drives fibrotic progression through coordinated dysregulation of lipid, amino acid, carbohydrate metabolism, and the TCA cycle.

Lipid metabolism in PF is characterized by impaired surfactant production and ectopic lipid accumulation. Alveolar type II (ATII) cells downregulate lipid synthesis genes (ABCA3) and cholesterol metabolism genes, reducing surfactant and promoting cholesterol deposition^63^-^66^. Loss of mitochondrial or endoplasmic reticulum proteins (mitofusins, EMC3) further disrupts lipid homeostasis^67^,^68^. Foam macrophages exhibit a pro-fibrotic phenotype via CD36-mediated fatty acid uptake, while inhibiting CD36 alleviates fibrosis^69^-^71^. Key lipid mediators regulate fibrosis. LTB4 enhances collagen deposition (blockade reduces fibrosis), PGE2 inhibits fibroblast proliferation (reduced in advanced PF), and PGF2α drives fibrosis independently of TGF-β 72-74.

Amino acid metabolism supports collagen biosynthesis in PF. TGF-β upregulates glycine synthesis, glutaminase 1, and α-KG to stabilize collagen^75^-^77^. Upregulation of arginase 1 in IPF drives proline synthesis, promotes collagen accumulation, and targeting this reduces fibrosis^78^,^79^. Asymmetric dimethylarginine (ADMA) accumulates due to dimethylarginine dimethylaminohydrolase (DDAH) inhibition, impairs NO production, and promotes fibrosis^80^.

Hypoxia stabilizes HIF-1α, enhances glycolysis and GLUT1 expression, and promotes fibrosis 70. TGF-β upregulates GLUT1 and LDH, increases lactate production; acidic pH activates TGF-β-mediated myofibroblast differentiation^81^-^83^. Overexpressed hexokinase/LDH in IPF fibroblasts support fibrosis, and hexokinase inhibition (e.g., lonidamine) has anti-fibrotic effects^81^,^84^.

The TCA cycle is disrupted, altering bioenergetics and epigenetics. Mitochondrial acetyl-CoA reduces histone acetylation, inhibits COX-2/PGE2, and promotes fibrosis^85^. Glutamine-derived α-KG activates mTORC1 to stabilize collagen and modulates anti-apoptotic XIAP via Jumonji domain-containing protein D3 (JMJD3)/ubiquitously transcribed tetratricopeptide repeat, chromosome X (UTX) to maintain fibroblasts 77,86.

### 2.6 Microbiome Dysbiosis​

Microbiota dysbiosis plays a key role in the progression of PF through the gut-lung axis and local lung ecology. Its pro-fibrotic mechanisms involve multidimensional pathways such as immune imbalance, metabolite signaling, and epithelial barrier damage.

At the gut-lung axis level, structural dysbiosis of the gut microbial community is a key driver of PF. Specifically, the reduction of Akkermansia muciniphila, which has immunomodulatory functions, disrupts immune homeostasis, leading to increased levels of systemic pro-inflammatory cytokines (such as IL-6, IL-17A) and suppression of anti-inflammatory cytokines (such as IL-10), thereby exacerbating lung inflammation and fibrosis via the circulatory system^87^. Additionally, the gut microbiota promotes M2 polarization of macrophages and Th cell imbalance by downregulating short-chain fatty acids and upregulating lipopolysaccharide (LPS), and accelerates PF progression by targeting core PF pathways (TGF-β, YAP/TAZ, metabolic reprogramming)^88^.

In the local lung, microbiome dysbiosis is also crucial, typically characterized by the enrichment of Gram-negative bacteria (e.g., Proteobacteria). LPS released by these bacteria activates Toll-like receptor 4 (TLR4) signaling, driving the TLR4/STAT3 phosphorylation cascade to promote Th17 cell differentiation and expression of the pro-fibrotic factor TGF-β1^99^,^100^. Environmental factors (such as PM2.5, silica) can disrupt lung microbial homeostasis and induce abnormal miRNA expression, exacerbating dysbiosis via the MAPK/IL-17 pathway and forming a feedforward loop that accelerates fibrosis^89^,^90^.

Insufficient expression of antimicrobial peptides due to primary immunodeficiencies (such as TLR5 deficiency), as well as sex differences, also modulate microbiome balance and affect PF susceptibility^91^,^92^.

## 3. Mechanisms by Which PFME Promotes Lung Carcinogenesis​

**Figure 4:**
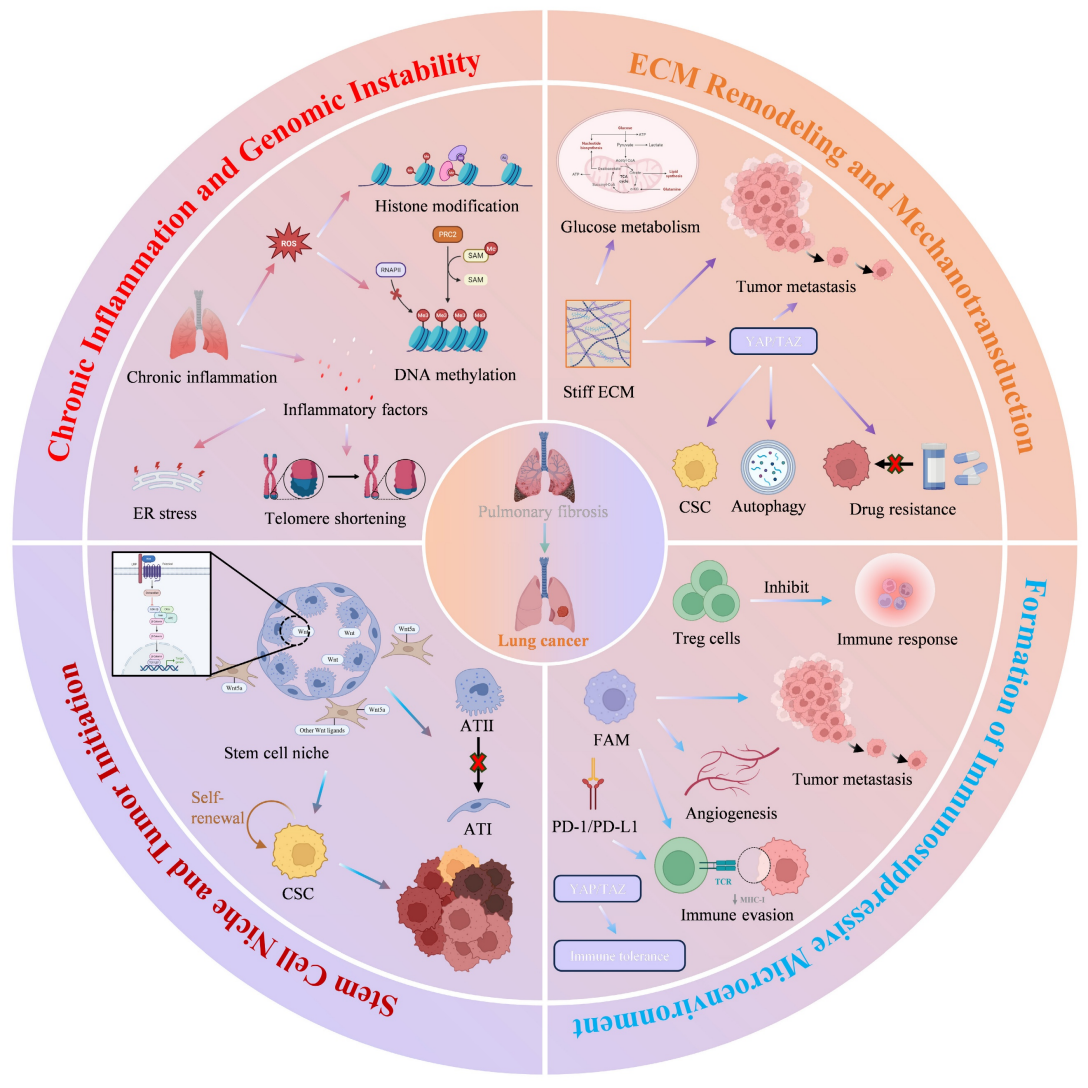
** Schematic diagram of the mechanism by which PFME promotes lung carcinogenesis.** Chronic inflammation, as a core pathological feature of PF, induces genomic instability through multidimensional mechanisms, providing a critical driving force for the malignant transformation of lung epithelial cells. ECM remodeling increases matrix stiffness, activates mechanical signaling, and reprograms glucose metabolic pathways in tumor cells, thereby promoting malignant progression, drug resistance, and metastasis of cancer. The formation of an immunosuppressive microenvironment exerts a significant impact on the malignant progression of cancer. The aberrant expression of Tregs, M2-type macrophages, and the immune checkpoint PD-1/PD-L1 in PFME contributes to the formation of an immunosuppressive microenvironment, influencing cancer progression. ATII lung stem cells exhibit active Wnt signaling and form a Wnt signaling niche. The Wnt niche drives the proliferative potential and disease progression of lung adenocarcinoma. Copyrighted from the BioRender platform.

The process by which the PFME drives lung cancer development is a complex, multidimensional, multi-stage synergistic process, and its core mechanism is illustrated in Figure 4 (Fig. 4). As the key pathological basis for the progression from PF to lung cancer, PFME reshapes the pulmonary microenvironment and drives malignant transformation through four core links. First, chronic inflammation, as an initiating factor, induces genomic instability via ROS-mediated oxidative stress, immunosuppression, and endoplasmic reticulum stress, laying the "initiation" foundation for the malignant transformation of alveolar epithelial cells. Second, ECM remodeling and mechanotransduction, by sensing changes in matrix stiffness through the YAP/TAZ signaling pathway, drive tumor cell proliferation, drug resistance, metabolic reprogramming, and immune escape, dominating the "progression-metastasis" stage. Third, construction of an immunosuppressive microenvironment, relying on the synergistic effects of Tregs, M2-polarized tumor-associated macrophages (TAMs), and the PD-1/PD-L1 axis, weakens anti-tumor immune responses and assists tumor "colonization-immune escape". Fourth, abnormal regulation of the ATII stem cell niche, which maintains stem cell stemness and drives malignant transformation via Wnt signaling, providing "seed" cells for lung cancer occurrence. These mechanisms do not exist in isolation but rather through signal crosstalk and stage overlap, collectively constituting a complete pathological network of PFME-promoted lung cancer occurrence. The following will focus on the above core links and analyze their molecular mechanisms and functional effects one by one.

### 3.1 Chronic Inflammation and Genomic Instability

Chronic inflammation, as a core pathological feature of PF, induces genomic instability through multidimensional mechanisms, providing a key driving force for the malignant transformation of alveolar epithelial cells during the initiation and promotion stages of lung cancer development. Chronic inflammation mediates oxidative stress and immunosuppression via ROS, laying the histological foundation for the malignant transformation of alveolar epithelial cells.

In the PFME, chronic inflammation continuously promotes the generation of ROS and inflammatory mediators^93^. ROS generated by cells drives multidimensional pathological processes through oxidative stress, including DNA damage, abnormal activation of signaling pathways, epigenetic alterations, cell phenotype transformation, and metabolic reprogramming, ultimately leading to the occurrence and progression of pulmonary fibrosis and lung cancer^32^,^94^. ROS generated by cells triggers oxidative stress: on one hand, it directly induces epithelial cell apoptosis, activates pro-fibrotic signaling pathways (such as TGF-β/MAPK), upregulates the synthesis of pre-fibrotic cytokines, generates myCAFs, and causes lung tissue damage and lays the "foundation" for fibrosis; on the other hand, excessive ROS alters DNA methylation patterns and regulates specific histone modifications, leading to abnormal gene expression profiles and driving multi-stage carcinogenesis progression^32^. For example, ROS can induce p53 mutations, opening the "gate" for cellular malignant transformation^33^. Additionally, ROS damages mitochondrial DNA and the respiratory chain, resulting in reduced ATP synthesis and increased ROS, forming a vicious cycle^95^. In terms of metabolism, ROS induces the Warburg effect via HIF-1α stabilization to support the energy demands of tumor cells^94^. Excess ROS leads to immune cell dysfunction by oxidizing key immune molecules and inhibits signal presentation between dendritic cells (DCs) and T cells^95^. ROS also drives M1 macrophage polarization, secreting TNF-α and IL-1β to exacerbate inflammation, while inhibiting M2 macrophage function and hindering ECM degradation^96^. Meanwhile, inflammation impairs DNA repair mechanisms, further exacerbating genomic instability and providing a molecular basis for subsequent clonal selection^97^.

Notably, PF-specific endoplasmic reticulum (ER) stress forms a positive feedback loop with inflammatory signals, amplifying the risk of genetic damage during the initiation stage. In PF, oxidative stress in the ER leads to protein misfolding and promotes ER stress, further leading to excessive ROS^98^. ROS induced by ER stress causes DNA double-strand breaks; under its sustained action, the function of the DNA-dependent protein kinase catalytic subunit (DNA-PKcs)-non-homologous end joining (NHEJ) pathway is inhibited, reducing the efficiency of DNA double-strand break repair, and leading to the accumulation of genomic instability and mutation burden^99^. Furthermore, when ROS accumulation exceeds the repair capacity, markers of genomic instability such as telomere shortening accelerate clonal selection during the promotion stage. Telomere dysfunction synergizes with chronic inflammation to accelerate genomic collapse, promoting clonal expansion during the promotion stage. Telomere shortening is driven by mutations such as telomerase reverse transcriptase (TERT)/ telomerase RNA component (TERC)^100^-^102^. Telomere shortening promotes IFN-γ secretion by activating the cyclic guanosine monophosphate-adenosine monophosphate synthase (cGAS)-stimulator of interferon genes (STING) pathway, which not only enhances the inflammatory microenvironment but also remodels cell cycle regulation via interferon signaling, forcing cells to accelerate clonal selection and expansion under the "stress" of genomic instability^103^.

### 3.2 ECM Remodeling and Mechanotransduction​

In the PFME, ECM remodeling and mechanotransduction drive the escalation of malignant cellular phenotypes through the YAP/TAZ signaling pathway during the progression and metastasis stages of lung cancer development.

As transcriptional co-activators, YAP/TAZ regulate key pathways in cytoplasm-nucleus shuttling: they form complexes with TEA domain family members (TEAD), bind to distal enhancer regions, promote histone acetylation and recruitment of transcriptional machinery, and target the regulation of cell cycle and anti-apoptotic pathways, providing molecular support for tumor cell proliferation^104^,^105^. In fibrotic lung tissue, spindle-shaped myofibroblasts highly express TAZ, and their pro-fibrotic effects are partially mediated by the transcriptional target plasminogen activator inhibitor-1 (PAI-1), which is not regulated by matrix stiffness and independent of TGF-β signaling, creating a "mechanical microenvironment" for tumor cell invasion^7^.

After cells sense increased ECM stiffness via integrins, the Rho-ROCK pathway mediates increased F-actin tension, promoting YAP/TAZ nuclear translocation and activation; this process is independent of the Hippo pathway, and YAP/TAZ still respond to ECM stiffness even in the absence of LATS1/2^6^,^106^,^107^. More critically, YAP/TAZ drive malignant progression by sensing ECM mechanical signals. They maintain cancer stem cell (CSC) traits and increase basal autophagy levels, thereby enhancing chemoresistance of cancer cells^108^,^109^. YAP/TAZ also promote the progression and metastasis of lung cancer in vivo^110^. YAP/TAZ bind to TEAD to directly activate PD-L1 gene transcription and secrete chemokines such as C-C motif chemokine ligand 2 (CCL2) and CCL5, attracting MDSCs and Tregs, suppressing CD8+ T cell function, and promoting the expression of T cell exhaustion markers (PD-1, TIM-3)^111^. Studies have shown that increased ECM stiffness reprograms the glucose metabolism of tumor cells and coordinates intercellular metabolic communication, thereby enhancing tumor activity^112^. Additionally, intrafibrillar crosslinks in the ECM may contribute to lung cancer metastasis^113^. In summary, the ECM-mechanical signaling axis comprehensively drives the evolution of lung cancer from local progression to distant metastasis, ranging from "mechanical microenvironment construction" to "metabolic-immune reprogramming."

### 3.3 Formation of the Immunosuppressive Microenvironment​

The synergistic effects of Tregs, M2-polarized TAMs, and the PD-1/PD-L1 axis construct an immunosuppressive microenvironment during the colonization and immune escape stages of lung cancer development.

Tregs exhibit a "stage-specific functional switch" in the progression of IPF: in the early stage, they drive EMT and promote fibrosis by secreting factors such as PDGF and TGF-β, providing a "fibrotic soil" for tumor cell colonization; in the late stage, they indirectly maintain lung homeostasis by enhancing epithelial repair and inhibiting excessive fibroblast accumulation^114^,^115^. Flow cytometry and single-cell sequencing confirm that in advanced PF, the proportion and hyperfunction of Tregs increase. Their accumulation, by secreting cytokines such as IL-10 and TGF-β, promotes the polarization of alveolar macrophages into the M2 type and inhibits effector T cell activation, while also inducing CD8+ T cell exhaustion, thereby forming an immunosuppressive microenvironment conducive to lung cancer growth^116^,^117^.

YAP/TAZ further enhance immune tolerance by recruiting MDSCs, while promoting TAMs to secrete IL-6 to enhance immunosuppression, forming a "fibrosis-immunosuppression" vicious cycle^111^. As key effector cells in tumor progression, M2-polarized TAMs in PF promote tumor angiogenesis by secreting VEGF, inhibit the activity of T cells and natural killer (NK) cells, express PD-L1/PD-L2 and recruit Tregs, assisting tumor cell colonization and growth from both vascular support and immune escape aspects^42^.

**Figure 5:**
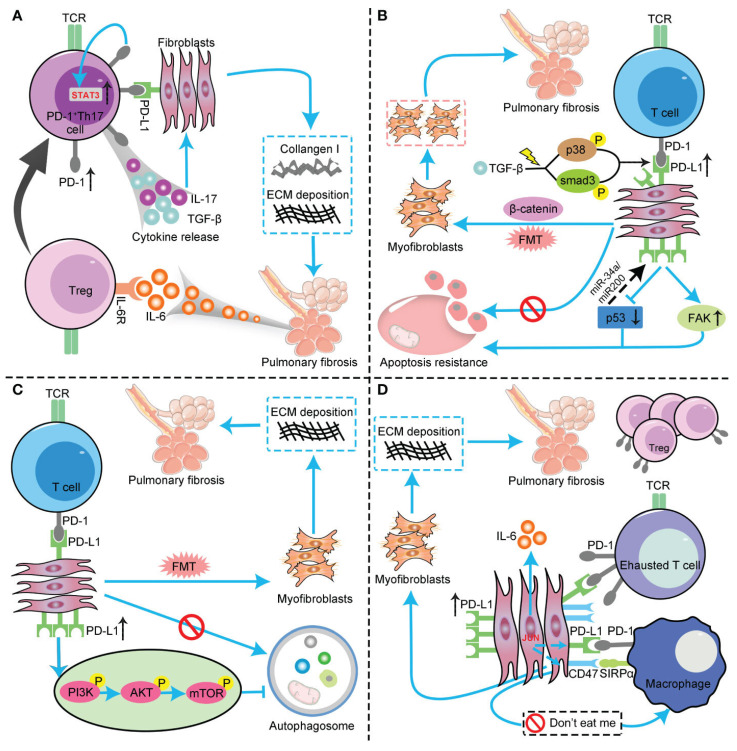
** The profibrotic role of the PD-1/PD-L1 axis in IPF through interaction with multiple cell types and pathways. (A)** PD-L1 up-regulation on Th17 T cells promotes pulmonary fibrosis through STAT3-mediated IL-17 and TGF-β production; **(B)** PD-L1 up-regulation on lung fibroblasts promotes pulmonary fibrosis *via* p53, FAK, Smad3, and β-catenin signaling pathways. On the one hand, PD-L1 up-regulation on lung fibroblasts may cause myofibroblasts to apoptosis-resistance and evasion phagocytosis *via* macrophages by inhibiting the p53 pathway and activating the FAK pathway, ultimately leading to excessive proliferation of myofibroblasts to trigger IPF. On the other hand, PD-L1 mediates lung fibroblast to myofibroblast transition (FMT) through Smad3 and β-catenin signaling pathways, thus promoting pulmonary fibrosis; **(C)** PD-L1 up-regulation on lung fibroblasts could induce myofibroblasts proliferation and ECM deposition through inhibiting autophagy, and eventually promotes pulmonary fibrosis; **(D)** PD-L1 up-regulation on lung fibroblasts promotes pulmonary fibrosis by inhibiting adaptive immunity. JUN upregulates the expression levels of PD-L1 and CD47 in fibroblasts and dormant macrophages. As a result, the above cells are converted into exhausted T cells and quiescent macrophages. In this context, myofibroblasts can evade immune clearance and resist macrophage-induced phagocytosis. In addition, JUN can also directly regulate IL-6 at the chromatin level, leading to inhibitory adaptive immune responses— primarily T cell exhaustion and upregulation of activated Tregs. FAK, focal adhesion kinase; TCR, T cell receptor; AKT, protein kinase B; PI3K, phosphoinositide 3-kinase; mTOR, mammalian target of rapamycin. (Note. From Jiang et al.^120^ (2022) under the terms of the Creative Commons Attribution International License (CC BY 4.0).).

Dysregulation of the PD-1/PD-L1 axis plays a central role in immune escape. The proportion of PD-L1-positive tumor cells is positively correlated with tumor severity and prognosis^118^. In IPF patients, the expression of PD-1/PD-L1 is abnormally elevated in lung tissues and peripheral blood. The mechanism is that the PD-1/PD-L1 signal inhibits excessive T cell activation under physiological conditions to maintain immune tolerance^119^-^121^(Fig. 5). However, in the TME, cancer cells upregulate PD-L1 to inhibit excessive T cell activation and cytokine secretion, and evade cytotoxic attacks by CD8+ T cells^122^,^123^. The activation of the PD-1/PD-L1 checkpoint in the PFME synergizes with PF-driven expansion of Tregs and M2 TAMs, weakening anti-tumor immune responses during the immune escape stage and clearing obstacles for the survival and proliferation of lung cancer cells.

### 3.4 Stem Cell Niche and Tumor Initiation​

Abnormal regulation of the ATII stem cell niche provides the "seed" for the formation of CSCs during the initiation stage of lung cancer development.

All stem cells, including ATII stem cells, are regulated by their local microenvironment or niche^124^. In normal alveolar repair, transient activation of Wnt signaling promotes ATII differentiation; while in the PFME, continuously activated Wnt signaling locks stem cell stemness and drives malignant transformation. Normal alveolar epithelial repair relies on the differentiation of ATII into ATI, but Hippo pathway inactivation in the PFME can elevate nuclear YAP/TAZ levels, enhance the alveolar regenerative capacity of the ATI cell lineage, and instead reduce bleomycin-induced fibrosis^10^. Conversely, excessive Hippo pathway activation drives aberrant differentiation of ATII stem cells, disrupts the homeostasis of alveolar epithelial repair, and increases the risk of PF and lung cancer^10^,^125^.

Adjacent mesenchymal fibroblasts of ATII stem cells secrete ligands such as Wnt5a to form a single-cell-scale Wnt signaling niche, preventing ATII stem cells from differentiating into ATI and maintaining their stemness^125^. This stemness maintenance mechanism acts as a "pusher" for tumor initiation when aberrant (e.g., Wnt/β-catenin signaling dysregulation). The Wnt pathway is a core pathway for stem cell self-renewal and carcinogenesis. Dysregulated Wnt/β-catenin signaling promotes the renewal, proliferation, and differentiation of cancer stem cells, playing a key role in tumorigenesis and therapeutic responses^126^. Studies have shown that aberrant activation of the Wnt pathway can promote the renewal or proliferation of colorectal cancer stem cells^127^-^129^. In lung cancer, however, it manifests as "malignant transformation of the alveolar stem cell niche": its Wnt-producing microenvironment drives enhanced stemness and increased proliferative potential of lung adenocarcinoma cells, while small-molecule inhibitors targeting key Wnt post-translational modifications can reduce tumor growth and significantly decrease lung cancer cell proliferation^128^. The Wnt/β-catenin pathway serves as one of the core mechanisms of lung cancer immune escape by inhibiting T cell infiltration, recruiting immune cells, and metabolic regulation^130^. This suggests that aberrant stem cell niche is a key link in lung cancer initiation, and disrupting this pathway may simultaneously intervene in fibrosis and tumorigenesis.

## 4. Impact of LC Progression on the PFME​

**Figure 6:**
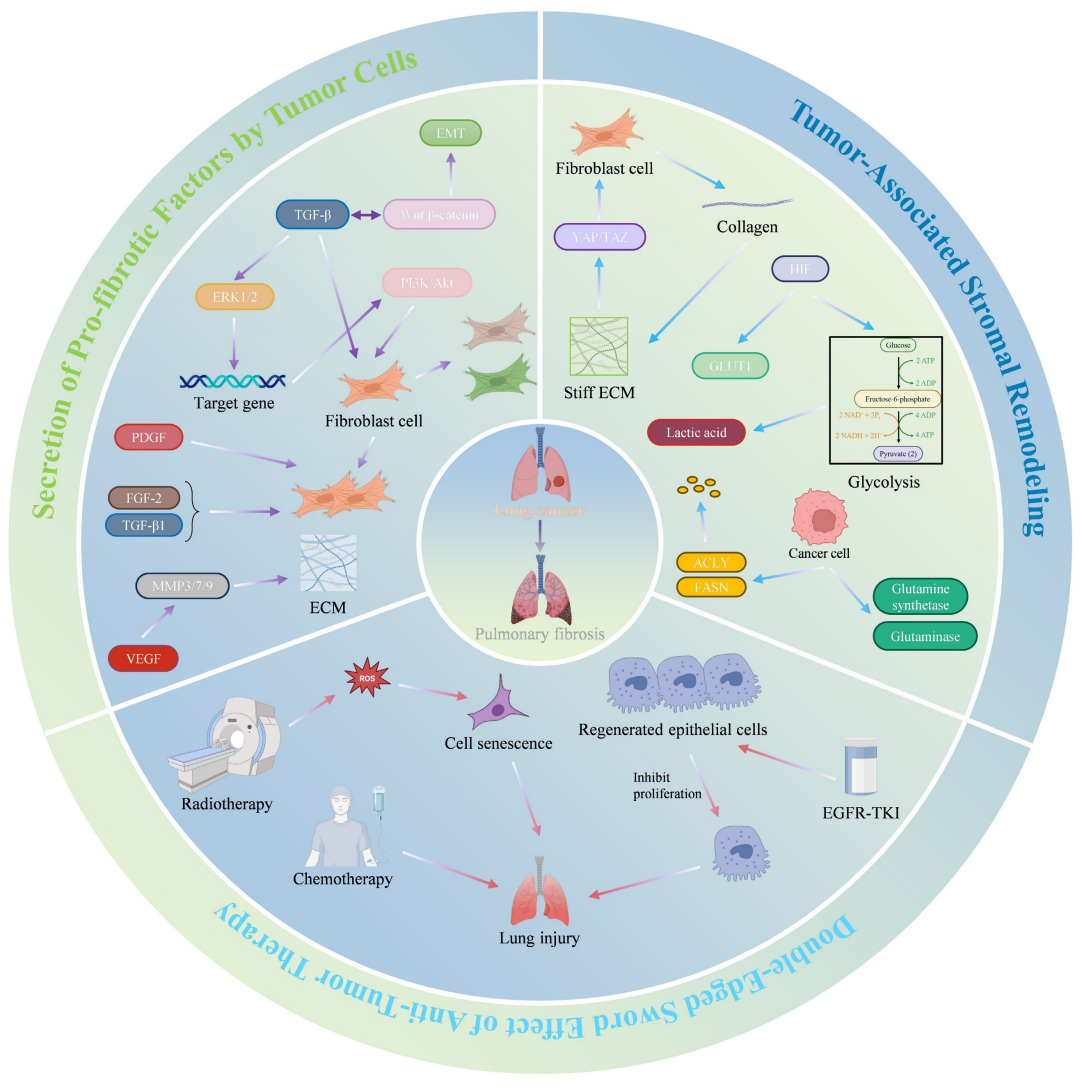
** Schematic diagram of the impact of LC progression on the PFME.** Crosstalk between the Wnt/β-catenin pathway and TGF-β can stimulate the proliferation and differentiation of myofibroblasts. Meanwhile, tumor cells secrete pro-fibrotic factors such as PDGF, VEGF, and FGF. Tumor-associated stromal remodeling can promote the progression of PF. Increased ECM stiffness activates the YAP/TAZ pathway, driving fibroblast activation and fibrosis, thus forming a feedback loop. Metabolic changes in tumor cells also promote PF progression. Additionally, tumor-targeted therapies, such as radiotherapy, chemotherapy, and targeted therapy, may also increase the potential risk of exacerbating PF. Copyrighted from the BioRender platform.

The progression of LC not only drives malignant transformation but also reversely remodels the PFME, creating a vicious cycle that exacerbates both diseases. This section details how LC progression impacts PFME across three key stages: First, during the tumor growth phase, cancer cells secrete pro-fibrotic factors and remodel the stroma; second, during the invasion-metastasis phase, which is characterized by ECM stiffening, mechanotransduction, and metabolic reprogramming; finally, during the therapeutic intervention phase, radiotherapy, chemotherapy, and targeted therapy may induce or exacerbate PFME. These effects collectively amplify PFME abnormalities, which in turn fuel further LC progression (Fig. 6).

### 4.1 Secretion of Pro-fibrotic Factors by Tumor Cells​

Lung PF is closely linked to the Wnt/β-catenin signaling pathway. This pathway regulates molecules associated with tissue invasion, such as matrix metalloproteinases (MMPs), laminins, and cyclin D1, which drive EMT. However, the most critical function of the Wnt/β-catenin pathway may lie in its crosstalk with TGF-β^131^. Aberrant activation of this pathway has been observed in cancers such as lung cancer and mesothelioma^132^. Specifically, TGF-β may activate extracellular signal-regulated kinases 1 and 2 (ERK1/2), while its target genes activate other signaling pathways, including the phosphatidylinositol 3-kinase (PI3K)/protein kinase B (Akt) pathway, which regulates proliferation and apoptosis. The role of PI3K in TGF-β-induced proliferation and differentiation of myofibroblasts has been demonstrated^133^.

Furthermore, tyrosine kinase receptor ligands, including PDGF, VEGF, and fibroblast growth factor (FGF), are abnormally expressed in lung cancer and PF^134^. PDGF signaling plays a critical role in tumor growth, angiogenesis, and lymphangiogenesis in cancer^135^. For instance, studies using A549 cells as a NSCLC model revealed that crenolanib (a PDGF receptor inhibitor) dose-dependently suppresses cell proliferation and induces apoptosis^136^. PDGF also regulates VEGF expression via autocrine mechanisms in NSCLC and facilitates CAF recruitment to tumor masses^135^,^137^. Beyond its angiogenic role, VEGF directly acts on cancer cells to modulate the growth, migration, and production of pro-angiogenic factors^138^. FGF influences NSCLC cell proliferation, therapeutic sensitivity, and apoptosis^139^.

Lung cancer cells promote PF progression by secreting tyrosine kinase receptor ligands such as PDGF, VEGF, and FGF—pro-fibrotic factors implicated in PF. Evidence indicates that dysregulated PDGF expression plays a pivotal role in PF development. In bleomycin-induced PF models, PDGF-A/B/C mRNA expression is significantly upregulated^140^,^141^. PDGF stimulates lung fibroblast proliferation, thereby driving PF progression^142^. TGF-β1 enhances PF by inducing FGF-2 release and upregulating FGFR1/2, while FGF-2 synergizes with TGF-β1 to promote fibroblast proliferation in IPF patients^142^-^144^. VEGF orchestrates extracellular matrix remodeling by inducing matrix metalloproteinase (MMP3/7/9) expression, and anti-VEGF gene therapy attenuates inflammation and fibrosis in bleomycin models^145^,^146^. Collectively, these tumor-derived pro-fibrotic factors synergistically advance PF progression, suggesting that targeting these factors may represent a promising therapeutic strategy.

Collectively, these tumor-derived pro-fibrotic factors act as key drivers of PFME fibrosis during the tumor growth phase, primarily via Wnt/β-catenin-TGF-β crosstalk and PI3K/Akt signaling to activate myofibroblasts.

### 4.2 Tumor-Associated Stromal Remodeling​

The stromal components within the TME undergo remodeling to promote tumor progression. Interactions between lung cancer cells and fibroblasts have been extensively reported in previous studies. Two primary subtypes of CAFs—myofibroblast CAFs (myoCAFs) and inflammatory CAFs (iCAFs)—have been described in multiple cancer types^147^,^148^. Analysis of human tissues revealed that α-SMA-positive myoCAFs correlate with poor prognosis across various cancers and resistance to immunotherapy in NSCLC^148^,^149^. ECM production and remodeling represent critical functions of fibroblasts in all tissues^150^. Tumor-driven activation of the Wnt/β-catenin pathway mediates TGF-β crosstalk, stimulating myofibroblast proliferation and differentiation, promoting collagen deposition, and increasing ECM stiffness^131^. Additionally, pro-fibrotic factors secreted by lung cancer cells, such as PDGF, VEGF, and FGF, further enhance myofibroblast proliferation and differentiation^134^. Myofibroblasts reciprocally drive malignant progression by secreting collagen, increasing ECM stiffness, and activating the YAP/TAZ pathway. Studies have demonstrated that YAP/TAZ-mediated mechanotransduction activates fibroblast activation and fibrosis^7^. Furthermore, Smad3 facilitates CAF generation via macrophage-myofibroblast transformation^151^. The interplay between lung cancer cells and myofibroblasts in TME remodeling not only advances lung cancer progression but also promotes PF, highlighting potential therapeutic targets.

A hallmark of cancer is metabolic reprogramming. Tumor cells alter carbohydrate, lipid, and protein metabolism to support proliferation. Hypoxia, a defining feature of the TME, upregulates HIFs during hypoxic responses, enhancing glycolysis and upregulating GLUT1 to promote PF^61^,^152^. The hypoxic microenvironment also suppresses anti-tumor immunity via metabolites such as lactate^61^. Lactate's pro-fibrotic role—mediated by reduced extracellular pH to amplify TGF-β-driven myofibroblast differentiation—has been previously described^81^,^82^. Cancer cells increase de novo lipogenesis by upregulating enzymes such as ATP-citrate lyase (ACLY) and fatty acid synthase (FASN)^153^. Amino acid metabolism reshapes the TME and modulates immune cell function, either enhancing or inhibiting immune surveillance^154^. In tumor cells, enzymes associated with glutamine metabolism, such as glutamine synthetase and glutaminase, exhibit elevated expression levels correlated with tumor severity and poor prognosis^155^. Glutamine metabolism also impacts immune cell function within the TME, influencing immune surveillance dynamics^156^. While the effects of lipid and amino acid metabolic reprogramming on PF have been discussed earlier, these processes collectively accelerate PF development. Their interplay within TME components drives coordinated progression of both tumors and PF.

Thus, during the invasion-metastasis phase, tumor-stromal interactions and metabolic reprogramming collaboratively reshape PFME into a pro-fibrotic, pro-tumor niche.

### 4.3 Double-Edged Sword Effect of Anti-Tumor Therapy​

There are multiple treatment modalities for tumors, but some approaches, such as radiotherapy and chemotherapy, carry an increased risk of exacerbating PF. Radiotherapy remains a cornerstone of definitive (curative) and palliative treatment for many malignancies. Patients receiving definitive radiotherapy typically have locally advanced, surgically unresectable, or medically inoperable malignant tumors. For patients whose final treatment is surgery or chemotherapy, radiotherapy is often used as adjuvant/consolidation therapy. However, chemotherapy as an anti-tumor treatment frequently causes lung injury^157^. Approximately 9-30% of cancer patients receiving thoracic tumor radiotherapy develop radiation-induced PF, which may be associated with radiation-induced cellular senescence^158^. Radiation-induced cellular senescence is linked to free radical reactions. The presence of oxygen enhances free radical-mediated cellular damage due to the formation of various ROS. Free radicals/ROS disrupt the structure and function of DNA, lipids, and proteins through ionization and/or reactions, leading to metabolic and functional alterations that ultimately result in senescence or cell death^158^. Chemotherapy also induces lung injury, with its toxic effects generally dose-dependent^159^. The mechanisms of PF caused by chemotherapy are often drug-specific. For example, vincristine promotes the transdifferentiation of fibroblasts into myofibroblasts via the P38 and ERK signaling pathways, thereby inducing PF^160^. In cancer patients treated with cisplatin, apoptotic-resistant lung fibroblasts exist, and their anti-apoptotic mechanism is associated with casein kinase 2 (CK2)/X-ray repair complementing defective repair in Chinese hamster cells 1 (XRCC1)-dependent DNA repair activity, contributing to the development and progression of PF^161^.

Targeted therapy is also a viable option for tumors but may be linked to PF progression. EGFR mutation-positive lung cancer patients benefit from survival advantages with EGFR-tyrosine kinase inhibitors (EGFR-TKIs), yet EGFR-TKIs can cause lung injury^162^(Table 1). EGFR plays a critical role in epithelial cell maintenance; thus, EGFR-TKIs may impair epithelial cell growth and migration, alter cytokine expression, and induce the recruitment of inflammatory cells, leading to subsequent tissue damage. Suzuki et al. reported that gefitinib treatment enhances fibrosis in bleomycin-induced mice, with the mechanism related to reduced proliferation of regenerative epithelial cells^163^. This suggests that EGFR-TKIs should be used cautiously in cancer patients with PF.

These findings highlight that in the therapeutic intervention phase, radiotherapy, chemotherap or targeted therapy (e.g., EGFR-TKIs) inadvertently exacerbate PFME damage through ROS, cellular senescence, or epithelial injury.

In summary, LC progression impacts the PFME through core mechanisms across three distinct stages. During the tumor growth phase, LC secretes pro-fibrotic factors via Wnt/β-catenin-TGF-β-PI3K/Akt crosstalk to stimulate myofibroblast proliferation. During the invasion-metastasis phase, tumor stromal remodeling and metabolic reprogramming stiffen the matrix and amplify fibrosis. During the therapeutic phase, radiotherapy, chemotherapy, or targeted therapy induce ROS, senescence, or epithelial injury, leading to PFME deterioration. These effects form a feedback loop (Fig. 6). LC-driven PFME remodeling further promotes tumor growth and fibrosis, highlighting the bidirectional crosstalk between LC and PFME. This aligns with the preceding description, where PFME initiates LC, and LC progression conversely exacerbates PFME, jointly driving disease severity.

## 5. Clinical Significance and Translational Medicine Perspective​

### 5.1 Diagnostic and Prognostic Biomarkers​

The diagnostic and prognostic biomarkers for lung cancer and PF are well-defined; given that the interconnections and interactions between PF and lung cancer have been elucidated in the preceding content, their diagnostic and prognostic biomarkers are interrelated (Table 2).

#### 5.1.1 Association of Fibrosis-related Biomarkers with Lung Cancer Prognosis​

MMP-7 belongs to fibrosis-related biomarkers and is a secreted proteolytic enzyme that can degrade various ECM components and other substrates, thereby promoting tumor cell invasion and metastasis. Studies have shown that high expression of MMP-7 in lung adenocarcinoma is an independent predictor of the high incidence of air space diffusion in lung adenocarcinoma, and multivariate analysis has revealed that MMP-7 expression is associated with tumor behavior and poor prognosis^164^. Compared with non-neoplastic lung tissues, the mRNA expression level of MMP-7 is higher in NSCLC tissues, further suggesting the association between MMP-7 and lung cancer prognosis^165^. Statistically, the positive expression rate of MMP-7 in NSCLC tissues is 68.89%, which is significantly higher than 14% in normal lung tissues. Additionally, the mean microvessel density (iMVD) of MMP-7-positive lung cancer tissues is 46.2 ± 6.77, significantly higher than 30.8 ± 7.54 in the negative group (t = 9.641, P < 0.001), indicating that MMP-7 promotes tumor angiogenesis^166^.

Krebs von den Lungen-6 (KL-6) plays an important role in various types of ILDs and can be used to assess the fibrotic process and the severity of disease progression^167^. KL-6 is a mucin 1 (MUC1) typically expressed on the surface of human ATⅡ cells^168^. MUC1 is highly expressed in patients with lung adenocarcinoma and is associated with higher tumor infiltration. Furthermore, MUC1-EGFR collaborates with IL-6 by activating the nuclear factor kappa-B (NF-Κb) and MAPK pathways to regulate the stemness and paclitaxel resistance of lung adenocarcinoma^169^. Studies have indicated that serum KL-6 can serve as a biomarker for lung cancer treatment-related interstitial lung disease (TR-ILD), with high KL-6 levels (cut-off value: >436 U/mL) being an independent risk factor for severe TR-ILD^170^. Excessively high serum KL-6 concentrations in patients with IPF combined with lung cancer are associated with poor prognosis; after 24 months of Nintedanib treatment, most patients' pulmonary function parameters and serum KL-6 levels tend to stabilize^171^. Serum KL-6 levels are significantly elevated in lung cancer patients both with and without ILD. ILD has a significant impact on the prognosis of lung cancer patients. However, in patients without ILD, elevated KL-6 levels may still be associated with poor prognosis^172^. This suggests that KL-6 may act as a trans-disease state pan-fibrosis-tumor microenvironment activation biomarker, reflecting prognostic risk independent of the pathological background of ILD.

#### 5.1.2 Association of Tumor-related Biomarkers with PF

Carcinoembryonic antigen (CEA) is an important tumor marker for colorectal cancer and several other malignancies. The human CEA family comprises 29 genes, of which 18 are expressed; 7 belong to the CEA subfamily, and 11 belong to the pregnancy-specific glycoprotein subfamily^173^. A study systematically comparing serum CEA levels across patients with different diseases found that serum CEA levels showed a decreasing trend in patients with PF, pancreatic cancer, uremia, chronic obstructive pulmonary disease, colon cancer, Alzheimer's disease, rectal cancer, and lung cancer, indicating that elevated serum CEA levels are associated with both cancer and non-cancerous diseases^174^. Serum CEA levels are frequently elevated in patients with interstitial lung disease (ILD), and the risk of cancer development in ILD patients increases with rising serum CEA levels, suggesting an interconnection between CEA and PF^175^.

Cytokeratin 19 fragment (CYFRA21-1) is a keratin degradation product released from damaged alveolar epithelium and is primarily used as a tumor marker with significant diagnostic value for lung cancer. A prospective observational study revealed that serum CYFRA21-1 concentrations in IPF patients correlate with disease progression and mortality, and CYFRA21-1 is localized to the proliferative epithelium in IPF lung tissues, indicating that CYFRA21-1 serves as a marker of epithelial damage and renewal, potentially becoming an important biomarker for the prognosis and treatment of IPF patients^176^.

Additionally, PD-L1, an immune tumor marker, is an immune inhibitory molecule that suppresses T cell activation, leading to tumor progression^177^. One study found that PD-L1 expression is upregulated in the fibrotic lung tissues and serum of IPF patients; this upregulated PD-L1 increases vimentin levels by inhibiting vimentin ubiquitination, stimulates EMT of alveolar epithelial cells, and thereby induces PF^178^. In summary, these tumor-related markers may serve as prognostic indicators for PF in the future, potentially improving clinical diagnosis and treatment for patients.

### 5.2 Treatment Challenges and Strategy Optimization​​

#### 5.2.1 Efficacy and Safety of Immune Checkpoint Inhibitors in Fibrosis-Lung Cancer Patients​

Immune checkpoint inhibitors (ICIs) revitalize antitumor immune responses by disrupting inhibitory immune checkpoint molecules such as PD-1 and cytotoxic T lymphocyte antigen 4 (CTLA-4). Currently, there are two main types of approved immune checkpoint inhibitors: those targeting CTLA-4 and those targeting the PD-1 and its ligand PD-L1 pathway^180^. PD-1 can promote PF through the STAT3 pathway, and silencing PD-1 reduces PF^44^. PD-L1 increases vimentin levels by inhibiting vimentin ubiquitination, stimulates EMT of alveolar epithelial cells, and thus induces PF; silencing PD-L1 reduces PF^178^. Additionally, studies have confirmed the potential benefits of targeting the PD-1/PD-L1 pathway in treating PF and indicated that this pathway is associated with immune regulation^121^. This undoubtedly demonstrates the feasibility of using ICIs to treat PF.

Currently, clinical studies on ICIs in patients with fibrosis-lung cancer are relatively limited. In an Asian study, 6 patients with mild idiopathic interstitial pneumonia (IIP)-NSCLC received nivolumab treatment^181^. After 12 weeks of treatment, none of the 6 patients experienced drug-related non-hematological grade 3/4 or hematological grade 4 adverse events. Furthermore, no patients developed pneumonia of any grade, and 3 cases achieved partial response. This supports the use of ICIs in patients with mild fibrosis-lung cancer and warrants further exploration and development.

However, while enhancing normal immune responses, ICIs may also amplify the antitumor effects of cellular immunity, leading to immune tolerance imbalance and immune-related adverse events (irAEs). IrAEs can involve all organs throughout the body and are common; data from one study show that more than 80% of patients using ICIs developed irAEs in some systems^182^,^183^. In targeted therapy with ICIs for lung cancer, immune-related pneumonia must be considered. Studies have found that the risk of developing immune checkpoint inhibitor-related pneumonia (CIP) is higher in ILD patients using ICIs compared to those without ILD, although these studies indicate that CIP is not associated with increased mortality^184^-^186^. In summary, in addition to investigating whether ICIs can improve disease progression in IPF patients, future studies should also clarify their role in prognosis.

#### 5.2.2 Potential Anticancer Effects of Antifibrotic Drugs

Currently approved drugs for PF treatment, such as nintedanib and pirfenidone, also exhibit effects in lung cancer. Nintedanib is a multi-target tyrosine kinase inhibitor (TKI) that primarily alleviates inflammation and fibrosis in lung connective tissues by inhibiting multiple signaling pathways, including vascular endothelial growth factor receptor (VEGFR), FGFR, and platelet-derived growth factor receptor (PDGFR). Notably, TKIs are targeted drugs for cancer treatment; thus, nintedanib is also approved for use in combination with docetaxel as an effective second-line option for advanced NSCLC patients^187^. In a randomized phase III trial involving 243 chemotherapy-refractory advanced NSCLC patients with IPF, the results showed that the median progression-free survival was 14.6 months in the nintedanib plus chemotherapy group versus 11.8 months in the chemotherapy group, with median progression-free survival times of 6.2 months and 5.5 months, respectively, indicating that combination chemotherapy improves overall survival in NSCLC patients^188^.

Pirfenidone is a pleiotropic molecule that inhibits TGF-β, collagen synthesis, and fibroblast proliferation, and mediates tissue repair^189^,^190^. Miuri et al. observed that the incidence of lung cancer in IPF patients treated with pirfenidone was significantly lower than in the non-pirfenidone IPF patient group^191^. Additionally, experimental data indicate that the combination of pirfenidone and cisplatin may increase the mortality of CAFs and tumor cells in preclinical models of non-small cell lung cancer^192^.

#### 5.2.3 Optimization of Combination Therapy Strategies

Combination therapy of antifibrotic drugs with ICIs is beneficial. Studies have shown that in patients with IPF combined with squamous cell carcinoma, the addition of nintedanib can prevent atezolizumab-induced pneumonia or acute exacerbation of IPF^193^. Another study demonstrated that pirfenidone combined with PD-L1 inhibitors in treating lung cancer-fibrosis mouse models significantly delayed tumor growth, reduced PF, and improved mouse survival rates compared to PD-L1 inhibitors alone^194^. When using antifibrotic drugs combined with ICIs to treat lung cancer patients with PF, considering antifibrotic therapy followed by immunotherapy may be a favorable option. This is because ECM stiffness can reduce the efficacy of ICIs, whereas antifibrotic drugs can lower ECM stiffness, thereby enhancing the efficiency of T cell infiltration by subsequent ICIs^195^.

### 5.3 Key Therapeutic Targets and Intervention Strategies

#### 5.3.1 Strategies Targeting the Dual-Edged Sword Effect of TGF-β​​

TGF-β plays a dual role in the fibrotic and lung cancer microenvironments—suppressing inflammation in the early stage and driving fibrosis and immune escape in the late stage^3^. Given the pivotal role of TGF-β in fibrosis and tumor progression, along with its complex dual effects, developing precise intervention strategies that can circumvent its detrimental effects while harnessing its beneficial roles represents a critical focus and challenge in current research. TGF-β activates fibroblasts through the canonical Smad pathway, promoting collagen deposition, which is its primary role in PF. In the development and progression of lung cancer, however, TGF-β exhibits a dual-edged sword effect. In the early stages of tumorigenesis, TGF-β acts as a tumor suppressor by inhibiting proliferation and inducing apoptosis^196^. However, the immunosuppressive microenvironment regulated by TGF-β can indirectly promote tumor escape^197^. For example, TGF-β controls the development and function of the innate immune system by inhibiting NK cells and regulating the proliferation of macrophages, antigen-presenting DCs, and granulocytes. It also suppresses the differentiation of CTLs, Th1, and Th2 cells, and even modulates B cell proliferation and differentiation^198^.

Therefore, effective intervention in the TGF-β signaling pathway is crucial for the simultaneous management of lung cancer and PF. Galunisertib, an oral small-molecule inhibitor of TGF-β receptor I (TβRI) kinase, specifically downregulates SMAD2 phosphorylation to inhibit activation of the canonical pathway. However, prolonged continuous exposure to galunisertib leads to cardiotoxicity^199^. The Phase Ib/II trial of Galunisertib combined with Nivolumab showed that there were no dose-limiting toxicities in the Phase Ib, and common treatment-related adverse events (TRAEs) in the Phase II NSCLC cohort (n=25) were itching (36%), fatigue (32%), and decreased appetite (28%). There was only one case of Grade 3 immune-related encephalitis, no Grade 4/5 TRAEs, and safety was comparable to monotherapy^200^. Furthermore, the median overall survival of this trial was 11.99 months and the median progression-free survival was 5.26 months; compared with which, the efficacy was superior in the TGF-β high-expression group^200^. Additionally, some TβRI kinase inhibitors exhibit cardiotoxicity and cutaneous toxicity at high doses^201^. Thus, there is a need to identify precise interventions targeting TGF-β and its downstream pathways or multi-targeted drugs.

Second-generation PD-1/PD-L1 inhibitors, such as M7824 and SHR-1701, have been developed to simultaneously block PD-1/PD-L1 and TGF-β/TGF-βR. M7824 is a bifunctional fusion protein composed of a monoclonal antibody against PD-L1 fused to the extracellular domain of human TβRII. It specifically binds to both PD-L1 and TGF-β. Studies show that M7824 inhibits tumor growth and metastasis by activating both the innate and adaptive immune systems, with effects superior to those of anti-PD-L1 antibodies or TGF-β traps alone^202^. Furthermore, in vitro and in vivo treatment of NSCLC cells with M7824 reduces TGF-β1-mediated mesenchymal features, including expression of mesenchymal markers, proliferation inhibition, and chemoresistance^203^. Another study indicates that SHR-1701 overcomes resistance to PD-1/PD-L1 inhibitors in lung cancer patients caused by lymphocyte recovery disorders^204^. A phase I expansion trial has verified the safety and efficacy of M7824. Among 80 patients with advanced NSCLC who had failed platinum-based chemotherapy (and had not previously received immunotherapy), the total objective response rate (ORR) was 21.3%, with an ORR of 25% at the recommended phase II dose (1200 mg). Notably, in the PD-L1-high expression subgroup (≥ 80%), a significant response was achieved (ORR = 85.7%), with a total TRAE rate of 69% and no treatment-related deaths^179^. These results demonstrate that M7824 is tolerable and exhibits therapeutic potential in platinum-resistant NSCLC, particularly in patients with high PD-L1 expression.

#### 5.3.2 Matrix Stiffness Regulation: Lysyl Oxidase (LOX) Inhibitors and YAP/TAZ Inhibitors

Matrix stiffness is a core mechanical feature of the fibrotic-lung cancer microenvironment. Targeting ECM cross-linking enzymes (e.g., the LOX family) can reverse pro-tumorigenic mechanical signals. The LOX family comprises five members: LOX, LOXL1, LOXL2, LOXL3, and LOXL4. These are copper-dependent enzymes in the extracellular space that play a key role in ECM cross-linking but also have other intracellular functions associated with fibrosis and carcinogenesis^205^. LOX catalyzes collagen/elastin cross-linking, increasing matrix stiffness, which activates YAP/TAZ signaling to promote PF and lung cancer development^7^,^110^,^206^. LOXL2 promotes tumor cell survival and chemoresistance, regulates cell adhesion, motility, and invasion, and remodels the TME. Growing evidence indicates that high LOXL2 expression correlates with tumor grade, poor prognosis, and reduced survival^207^. Thus, the LOX family represents a promising target.

LOX inhibitors have been developed and are undergoing clinical trials. Simtuzumab, a monoclonal antibody against LOXL2, failed to improve lung function in IPF patients when used alone. In a phase II trial comparing simtuzumab with placebo in IPF patients, no difference in progression-free survival was observed between the two groups^208^. This may be related to simtuzumab's anti-angiogenic effects but lack of inhibition of LOXL2 catalytic activity, collagen cross-linking, or tissue stiffening^209^. Although simtuzumab cannot be used alone for PF treatment, its anti-angiogenic properties suggest potential synergistic effects when combined with nintedanib, though further experiments are required. In contrast, PXS-5505 is a potent inhibitor. PXS-5505 is a novel small-molecule pan-LOX inhibitor. In a bleomycin-induced mouse lung model, PXS-5505 normalized collagen/elastin cross-linking, reducing PF to normal levels^210^. Additionally, PXS-5505 decreases chemotherapy-induced connective tissue formation and stiffness in pancreatic cancer, with its mechanism of enhancing chemotherapeutic efficacy linked to reduced matrix stiffness^211^. These findings suggest that PXS-5505 can disrupt the ECM barrier to enhance drug efficacy, and it may be combined with PD-1 antibodies or other drugs in the future to synergistically enhance anti-fibrotic and anti-tumor effects.

The role of the YAP/TAZ pathway has been elucidated earlier and it is a potential therapeutic target. Verteporfin is a small-molecule inhibitor of YAP-TEAD that specifically interferes with the formation of the YAP-TEAD protein complex and can significantly suppress YAP-dependent malignant tumor phenotypes^111^. Verteporfin inhibits the proliferation of non-small cell lung cancer (NSCLC) cells and induces apoptosis by inhibiting the expression of Anoctamin 1 protein and blocking the EGFR-STAT3 signaling pathway, and it is effective against gefitinib-resistant cells with good safety^212^. IAG933 is also a YAP-TEAD inhibitor that demonstrates significant antitumor activity in various cancer models (including lung cancer), especially with better efficacy when combined with EGFR, Kirsten rat sarcoma 2 viral oncogene homolog (KRAS), or MEK inhibitors^213^. Although direct clinical trial data on YAP/TAZ inhibitors are relatively limited, the existing preclinical data has demonstrated that this pathway can effectively inhibit cancer.

#### 5.3.3 Synergistic Strategies of Exercise Intervention in Anti-Cancer and Anti-Fibrosis

Recent studies on exercise have consistently demonstrated its role in promoting health and combating cancer. As a non-pharmacological intervention, exercise training can reshape the TME and fibrotic microenvironment through multi-system regulation, exhibiting unique advantages in inhibiting tumor progression and fibrosis. First, in terms of prevention, exercise reduces the risk of lung cancer development; second, it improves patients' quality of life; third, it synergistically enhances the efficacy of other therapies. The biological basis of exercise in lung cancer anti-cancer intervention primarily includes regulating the tumor immune microenvironment, inhibiting tumor angiogenesis, promoting tumor cell apoptosis, and modulating the ECM^214^. Its anti-fibrotic effects mainly involve directly inhibiting fibroblast activation, reducing the expression of pro-fibrotic factors such as TGF-β, and slowing collagen deposition^215^.

**Figure 7:**
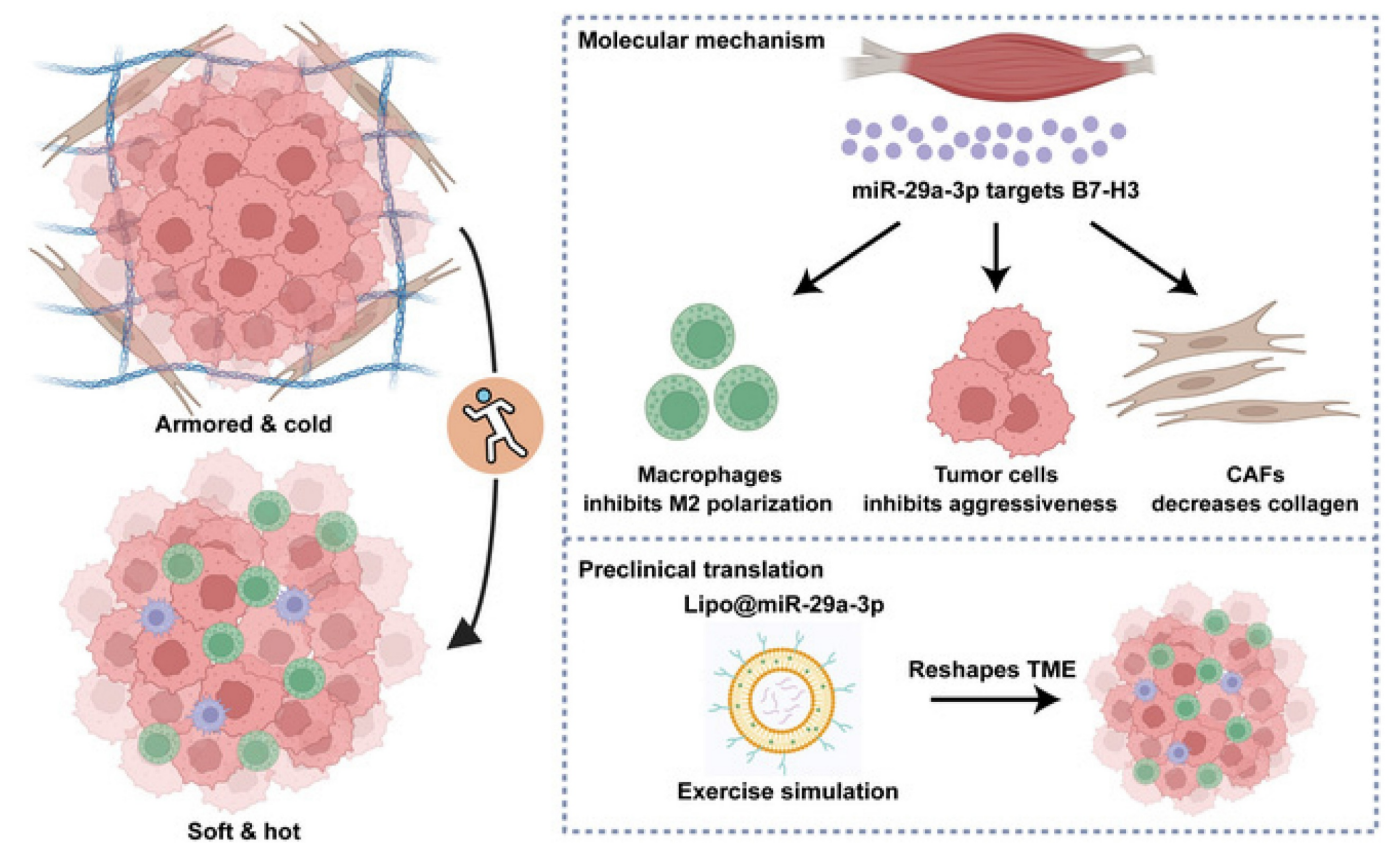
** Schematic overview of the current study.** Exercise-responsive miR-29a-3p can make armored and cold tumors turn into soft and hot tumors, which greatly increases its therapeutic significance in clinical practice. Mechanistically, miR-29a-3p targets B7-H3 expression to inhibit tumor cell aggressiveness, macrophage M2 polarization, and collagen synthesis of fibroblasts. Translationally, a novel biomaterial lipo@miR-29a-3p was developed to simulate the benefits of exercise. Copyrighted from the BioRender platform. (Note. From Mei et al.^217^ (2025) under the terms of the Creative Commons Attribution International License (CC BY 4.0).).

Exercise-induced miR-29a-3p can reduce ECM stiffness, increase T cell infiltration, and enhance immune response to therapy^216^. Studies have reported that miR-29a-3p is an exercise-responsive miRNA that inhibits B7 homolog 3 (B7-H3) expression to suppress macrophage M2 polarization, tumor cell invasiveness, and collagen synthesis by fibroblasts. Additionally, this study developed a novel biomaterial, lipo@miR-29a-3p, to mimic the benefits of exercise^217^(Fig. 7). Furthermore, exercise can regulate THSD7B expression, which exhibits positive prognostic implications and tumor-suppressive functions across cancers^218^. Objectives, safety, feasibility, and efficacy of exercise training in the context of early-stage and advanced lung cancer have been demonstrated. Exercise training in early-stage lung cancer patients (preoperative and postoperative) and advanced lung cancer patients can improve exercise tolerance, prevent complications, and enhance quality of life^219^,^220^. Studies have also shown that exercise serves as a rehabilitation strategy for PF patients, significantly improving their exercise capacity, lung function, and cardiorespiratory endurance^221^. Thus, integrating reasonable exercise into anti-cancer and anti-fibrotic strategies contributes to improved patient prognosis and quality of life.

In summary, the multi-dimensional regulatory roles of targeting TGF-β dual effects, matrix stiffness modulation, and exercise intervention collectively represent a new paradigm for fibrosis-lung cancer treatment. Exercise reshapes the tumor immune microenvironment, inhibits abnormal ECM remodeling, and modulates the tumor metabolic microenvironment, synergizing with targeted strategies. Combined with spatiotemporal selective inhibition, mechanoresponsive drugs, and intelligent combination strategies, these approaches are expected to break through current treatment bottlenecks and achieve a transition from "anti-fibrosis + anti-cancer" linear intervention to "microenvironment reversal + tumor eradication" systemic breakthroughs.

## 6. Discussion and Future Directions​​

### 6.1 Controversies and Unresolved Questions​​

#### 6.1.1 Does the Fibrotic Microenvironment Directly Induce Lung Cancer, or Act Only as a "Cancer-Promoting Soil"?​​

There is ongoing debate as to whether the PFME directly induces LC or merely acts as a "cancer-promoting soil." The "direct carcinogenesis" hypothesis is supported by genomic damage and epigenetic drivers. Chronic inflammatory factors in the PFME (e.g., IL-6, TNF-α) and ROS can directly induce TP53 gene mutations and DNA damage in alveolar epithelial cells 32,33,110. In metaplastic and bronchiolar epithelial samples from LC-IPF patients, allelic loss of the candidate tumor suppressor gene fragile histidine triad (FHIT) is more common than in IPF patients without LC^222^. Additionally, specific point mutations at codon 12 of the KRAS gene have been detected in LC-IPF patients^223^. Interestingly, this mutation has not been identified among the numerous KRAS mutations observed in lung cancer tissues^224^. Similar to IPF, miR-21 is overexpressed in LC and serves as an independent negative prognostic factor for overall survival in NSCLC patients^225^. These findings support the "direct carcinogenesis" hypothesis.

The "cancer-promoting soil" hypothesis is supported by arguments prioritizing immune escape and contradictory observations. Infiltration of Tregs and M2-type macrophages in the PFME weakens immune surveillance, allowing pre-existing premalignant cell clones to expand and promoting angiogenesis and metastasis in cancer cells^41^,^42^,^117^. Aberrant PD-1/PD-L1 expression in IPF patients suppresses excessive T cell activation and cytokine secretion to maintain immune tolerance to self-antigens, thereby assisting cancer cells in evading immune surveillance^120^,^122^,^123^. Furthermore, studies have found that due to promoter hypermethylation, the relative expression of SMAD4 (a known tumor suppressor gene) in LC tissues is significantly lower in LC-IPF patients compared to LC patients without IPF^226^,^227^. These studies suggest that the microenvironment may primarily promote the survival of mutated cells rather than directly inducing mutations.

Of course, it is also possible that both hypotheses coexist, with one predominating under certain conditions. Future research is needed to resolve this controversy to better understand the link between PF and LC and improve patient care. Future studies could construct humanized PF organoid models to clarify the oncogenic contributions of microenvironmental components via genetic editing (e.g., clustered regularly interspaced short palindromic repeats (CRISPR) knockout of ROS-generating enzymes). Clinically, longitudinal tracking of free DNA (cfDNA) mutation dynamics in the bronchoalveolar lavage fluid of PF patients could correlate with the timing of lung cancer onset. If direct carcinogenesis dominates, early genetic intervention strategies (e.g., antioxidants) should be developed; if the "cancer-promoting soil" hypothesis prevails, enhanced immune monitoring (e.g., regular assessment of CD8+ T cell function) should be prioritized.

#### 6.1.2 Heterogeneity of Lung Cancer Impact Across Different Types of Fibrosis​​

Different types of fibrosis exert distinct effects on lung cancer, primarily including IPF, autoimmune-related PF, and occupational pneumoconiosis. IPF is a chronic, progressive, fibrotic interstitial lung disease with lesions confined to the lungs, predominantly affecting middle-aged and elderly individuals. The prevalence of LC in IPF patients ranges from 2.7% to 48%, significantly higher than in the general population^134^ (Table 3). As previously discussed, IPF pathogenesis is primarily driven by TGF-β/Smad signaling, high ECM stiffness, and hypoxia. Autoimmune-related PF, exemplified by rheumatoid arthritis-associated interstitial lung disease (RA-ILD), is a PF linked to autoimmune processes. Studies indicate that RA is associated with a >50% increased risk of lung cancer, while RA-ILD patients represent a particularly high-risk group with a ~3-fold increased risk^228^. RA-ILD may arise from immune recognition of post-translationally modified proteins (induced by smoking or other lung injuries), leading to secondary joint disease characteristic of RA^229^. Additionally, RA-ILD results from environmental-genetic-immune interactions that expose modified antigens, generate anti-citrullinated protein antibodies (ACPA), and drive chronic inflammation, ultimately progressing to usual interstitial pneumonia (UIP)-like pathological changes^230^. Pneumoconiosis refers to a group of lung diseases caused by inhalation of respirable particles (typically < 5 μm in diameter) reaching the terminal airways and alveoli, with silicosis as a representative example. Silicosis is an occupational lung fibrosis characterized by diffuse nodular PF caused by long-term inhalation of free silica dust in occupational settings. Studies have reported a correlation between silicosis and increased lung cancer risk, with an odds ratio (OR) of 1.47^24^. The pathophysiology of silicosis involves silica particle deposition in alveoli. Macrophage phagocytosis of these particles triggers inflammation, stimulates fibroblast proliferation, and collagen production; additionally, silicosis is associated with autoimmunity^231^.

Given the distinct mechanisms of different fibrosis types, do differences in immune cell infiltration patterns lead to variations in pro-tumorigenic factors? Do the physical properties of particles in occupational PF influence lung cancer subtypes? Furthermore, do these differences impact clinical management? For example, anti-fibrotic drugs (e.g., nintedanib) are effective for IPF-associated LC but may exacerbate immune imbalance in autoimmune PF. Addressing these questions may require multi-omics comparisons and organoid model construction. Mechanistically, spatial transcriptomic sequencing of LC tissues from IPF, RA-ILD, and pneumoconiosis patients could identify subtype-specific pathways. Clinically, disease-specific PF-LC co-culture systems could screen for type-dependent therapeutic targets, facilitating the development of subtype-specific monitoring guidelines.

#### 6.1.3 Impact of Spatiotemporal Heterogeneity of the Fibrotic Microenvironment on Lung Cancer​​

The PFME is spatiotemporally heterogeneous, with molecular and cellular compositions varying across regions (spatial) and disease stages (temporal), forming a dynamically evolving pathological niche. Spatially, the fibrotic region can be divided into an active fibrotic frontier (activated fibroblasts with high collagen triple helix repeat containing 1 (CTHRC1)/FAP expression) and an end-stage fibrotic zone (extensive collagen deposition with low cell activity). Temporally, the progression spans from alveolar epithelial injury (loss of ATI cells, proliferation of ATII cells) to fibroblast activation, macrophage phenotype switching (fatty acid binding protein 4 (FABP4)+ to secreted phosphoprotein 1 (SPP1)+), and finally end-stage fibrosis^232^.

Mechanisms by which PFME spatiotemporal heterogeneity impacts LC development include EMT, immune microenvironment remodeling, and molecular gradient formation. Keratin 5 (KRT5) -/KRT17+ abnormal basal-like cells spatially co-localize with activated fibroblasts, promoting MMP7 and collagen, type I, alpha 1 (COL1A1) expression^233^-^235^. This enhances ECM remodeling, providing a physical scaffold for LC invasion and promoting EMT. SPP1+ macrophages are enriched during fibrotic progression, potentially activating pro-fibrotic and immunosuppressive pathways via TGF-β^236^,^237^, thereby inhibiting anti-tumor immunity and promoting LC immune escape. Pseudo-time trajectory analysis of alveolar regions from homeostasis to end-stage fibrosis reveals dynamic changes in transcription factors such as SRY-related high-mobility-group box 4 (SOX4)/SOX9^235^,^238^, which drive the maintenance of LC stem cell properties and enhance chemoresistance.

In summary, spatially, the active frontier zone primarily promotes invasion (via increased EMT and MMP production), while the end-stage zone primarily promotes hypoxia adaptation (via increased HIF-α factor production and angiogenesis). Temporally, early stages are driven by epithelial injury and clonal selection (KRAS/TP53 mutations), whereas late stages are dominated by immune suppression promoting metastasis (enrichment of SPP1+ macrophages)^232^. Could this spatiotemporal heterogeneity lead to differences in treatment response? From a spatiotemporal perspective, early-stage therapy should prioritize anti-fibrosis (e.g., nintedanib), mid-stage therapy should target fibroblasts (e.g., FAP inhibitors), and late-stage therapy should focus on immune combination approaches (e.g., anti-PD-L1 + anti-SPP1 drugs). Additionally, matrix degradation strategies (e.g., LOX inhibitors) should be considered in advanced stages. Future studies could utilize spatial multi-omics and dynamic imaging to analyze PFME spatiotemporal heterogeneity and develop location-targeted drug delivery systems.

In conclusion, resolving these controversies will advance PF-LC research from "phenomenological association" to "mechanistic intervention," providing a theoretical framework for personalized prevention and treatment.

### 6.2 Technological Drivers of Research Opportunities​​

Emerging technologies are redefining the research paradigm of pulmonary fibrosis-lung cancer microenvironment (PF-LCME) studies, transitioning from single-cell resolution to spatiotemporal dynamic dimensions and providing revolutionary tools for dissecting complex interaction networks.

#### 6.2.1 Single-Cell Sequencing and Spatial Multi-Omics​​

Single-cell sequencing and spatial multi-omics are currently playing pivotal roles. Single-cell multi-omics aids in resolving cellular heterogeneity. Single-cell RNA sequencing (scRNA-seq) has identified three distinct epithelial cell subtype populations in IPF, characterized by conducting airway basal cells and goblet cells, as well as additional atypical transitional cells that contribute to pathological processes in IPF^63^. Additionally, scRNA-seq has identified a highly enriched KRT5⁻/KRT17⁺ pathologically ECM-producing epithelial cell population in PF lungs^233^. Furthermore, single-cell analysis has revealed the role of FAP+ CAF (also called CAF-S1) in immunotherapy resistance^148^. Single-cell atlas analysis has identified distinct cell types in advanced NSCLC, with significant heterogeneity across patients in terms of cell composition, chromosomal structure, developmental trajectories, intercellular signaling networks, and phenotypic dominance^239^. Single-cell sequencing of LC and PF patients can determine potential intercellular interactions between the two conditions.

Spatial multi-omics facilitates the localization of interaction hotspots. Spatial transcriptomics has uncovered dysregulated molecular niches associated with distal lung remodeling in PF, informing other spatial transcriptomic studies^232^. One study combined spatial transcriptomics and scRNA-seq to reveal that spatially and transcriptionally distinct fibroblast lineages converge into transcriptionally consistent myofibroblasts, identifying secreted frizzled-related protein 1 (SFRP1) as a regulator of TGF-β1-driven fibroblast phenotypes during fibrogenesis^240^. This advances the development of therapeutic interventions to limit or reverse fibroblast focus formation. Future applications of spatial transcriptomics, spatial proteomics, and spatial metabolomics could reveal a ternary coupling of collagen deposition-lactate metabolism-immune suppression in fibrotic regions.

#### 6.2.2 Organoids and Co-Culture Models​​

Organoids are miniature organ models formed by self-organization of stem cells (e.g., pluripotent stem cells, adult stem cells) or tissue-derived cells under three-dimensional culture conditions, exhibiting structural and functional features similar to genuine organs. They are primarily applied in disease modeling, drug screening, and regenerative medicine. Co-culture involves culturing two or more cell types in the same system to simulate intercellular interactions (e.g., paracrine signaling, physical contact), with key applications in TME simulation, neural circuit construction, and host-pathogen interactions. The combined use of these two approaches is currently driving the development of high-fidelity disease models and precision medicine.

In PF-related research, Yao et al. analyzed interactions between primary fibroblasts and ATⅡ cells in organoid models^241^. They found that mucin 5B (MUC5B) was associated with ATⅡ cells in IPF patient samples. Their model demonstrated that fibrotic primary fibroblasts induce impaired differentiation of AT2 cells via the STAT3 signaling pathway, a phenomenon also observed in IPF patients. This model can be used for mechanistic studies and drug development. In cancer-related research, Esther et al. used co-cultures of colon cancer cells and CAFs to recapitulate the mesenchymal-like invasive characteristics of colorectal cancer^242^, aiding in understanding the relationship between CAFs and cancer cell invasiveness.

To better understand PF-LC interactions, future studies could use organoids and co-culture models to simulate the fibrosis-tumor interface. For example, constructing three-dimensional fibrosis-tumor organoids by co-culturing IPF patient-derived lung fibroblasts with lung cancer cells to recapitulate ECM stiffness and hypoxia gradients. Alternatively, developing fibrosis-tumor co-culture systems on chips to parallel-test 120 drug combinations for high-throughput drug screening.

#### 6.2.3 Artificial Intelligence (AI) Predictive Models​​

The advent of AI has brought numerous conveniences to scientific research. AI applications in medicine primarily branch into two categories: virtual and physical. The virtual branch includes informatics methods ranging from deep learning for information management to health management system control, as well as tools that actively guide clinicians in treatment decisions. The physical branch is best represented by robots that assist elderly patients or attending physicians, including nanoscale targeted robots—an innovative drug delivery system^243^.

High-resolution computed tomography (HRCT), a more sensitive imaging technique, is considered a core diagnostic tool for interstitial lung diseases^244^. Park et al. used AI to analyze HRCT lung segmentation in 647 ILD patients, achieving an accuracy rate of 98%^245^. Two studies demonstrated excellent performance in IPF diagnosis, with diagnostic accuracy approaching expert levels (78.9%)^246^,^247^. Additionally, Ren et al. used predictive AI methods to identify TRAF2 and NCK interacting kinase (TNIK) as an anti-fibrotic target and generated a small-molecule TNIK inhibitor, INS018_055^248^. AI also excels in lung cancer diagnosis and prognosis, with various models achieving diagnostic accuracy rates exceeding 70%, underscoring its value in these areas^249^.

Future applications of AI could involve analyzing HRCT images to extract fibrotic region texture features and constructing lung cancer risk models, using deep learning algorithms to automatically segment honeycombing regions and predict canceration probability. Furthermore, AI might integrate multi-omics data—for example, combining single-cell data, spatial transcriptomics, and clinical information to build heterogeneous networks, identify key driver nodes, and construct graph neural networks. AI could even dynamically predict changes in serum or other biomarkers to adjust treatment regimens.

In summary, the synergistic application of single-cell and spatial omics, organoid models, multi-omics integration, and AI is progressively unraveling the "black box" of the PF-LC microenvironment. Future efforts should focus on technological standardization, clinical translation validation, and ethical norms to advance these tools from the laboratory to personalized diagnostics and therapeutics, ultimately improving patient outcomes.

### 6.3 Translational Medicine Challenges​​

#### 6.3.1 Balancing the Conflicting Demands of Anti-Fibrotic and Anti-Tumor Therapies​​

The cross-application of anti-fibrotic therapy and anti-tumor therapy has emerged as a core direction in the comprehensive management of tumors in recent years. However, their complex interactions—the coexistence of synergy and antagonism—pose a key challenge in clinical practice. This dual characteristic necessitates breakthroughs in three dimensions: mechanistic elucidation, toxicity control, and strategy optimization—to drive the transformation of combined therapy from "empirical attempts" to "precision-designed" approaches.

The interaction between anti-fibrotic therapy and anti-tumor therapy is essentially a dynamic game involving multiple signaling pathways and cell types within the TME, with its efficacy highly dependent on the functional positioning and temporal characteristics of therapeutic targets in the TME. TGF-β acts as a "double-edged sword" in the TME: in the early stages of tumorigenesis, it exerts anti-tumor effects by inhibiting proliferation, inducing apoptosis, and suppressing cell immortalization; however, as the tumor progresses, its anti-tumor effects gradually diminish, and it instead promotes tumor deterioration through mechanisms such as EMT, migration, and immune suppression^250^. Consequently, while TGF-β inhibitors reduce fibrosis, they may also weaken the "natural anti-tumor" function of TGF-β in early-stage tumors. For example, in hepatocellular carcinoma models, TGF-β inhibitors, while alleviating fibrosis, may abrogate the early anti-tumor inhibitory effects of TGF-β, leading to rapid tumor progression^196^. This contradiction highlights that the clinical application of TGF-β inhibitors must be strictly restricted to "progressive tumors dominated by fibrosis" rather than early-stage or low-fibrosis tumors. Anti-angiogenic drugs reduce abnormal tumor angiogenesis by inhibiting VEGF signaling. Theoretically, they can improve drug delivery efficiency and enhance immune cell infiltration through "vascular normalization." However, excessive inhibition of VEGF damages normal vascular endothelial cells, inducing pulmonary hypoperfusion and chronic ischemia, which exacerbates tumor hypoxia, promotes EMT, and drives metastasis—as observed with bevacizumab^251^.

The combined application of anti-fibrotic and anti-tumor therapies requires attention to additive toxicity, which necessitates risk reduction through multi-dimensional strategies. ICIs exert anti-tumor effects by relieving T cell suppression, but their activation of the immune system may also attack normal tissues, inducing irAEs. Although studies have suggested that pirfenidone combined with ICIs is safe in patients with IPF and NSCLC, only four reported cases cannot fully confirm its safety^252^. The optimal approach is to evaluate the patient's pulmonary immune microenvironment status using multi-parameter biomarkers (e.g., serum KL-6, IL-6 levels in bronchoalveolar lavage fluid) before combination therapy to avoid a "1+1>2" toxic effect.

Optimizing the sequence of anti-fibrotic and anti-tumor therapies requires a foundation in the dynamic evolutionary characteristics of the TME, with the core focus being precise control over "when to intervene," "how to switch," and "how long to maintain." For patients with high fibrosis severity and tumors in a relatively quiescent stage, anti-fibrotic therapy should be prioritized. Anti-fibrotic drugs reduce ECM stiffness, minimize abnormal vascular leakage, and thereby improve subsequent anti-tumor efficacy. For "dual-driver" patients where fibrosis and tumor progression coexist, alternating use of anti-fibrotic and anti-tumor drugs can avoid long-term cumulative toxicity from single-agent therapy. The timing window for treatment sequencing must be adjusted based on the real-time status of the TME. Dynamic monitoring via imaging techniques (e.g., multi-organ ultrasound elastography to assess ECM stiffness) or molecular biomarkers (e.g., serum hyaluronic acid, fibroblast growth factor) is necessary to prevent loss of efficacy due to premature treatment switching.

The interaction between anti-fibrotic and anti-tumor therapies is essentially a microcosm of TME complexity. Their clinical application demands breaking through the linear "either-or" mindset and shifting to a "dynamic balance" systems perspective. In the future, with advancements in technologies such as single-cell sequencing and spatial transcriptomics, we aim to more precisely parse the interaction networks of various cell types in the TME. Combined with AI-driven treatment response prediction models, this will ultimately enable "personalized" precision combination therapy, representing not only a breakthrough direction for anti-tumor treatment but also the best practice of the translational medicine concept "from bench to bedside."

#### 6.3.2 Organ-Specific Drug Delivery Systems​​

Targeted delivery to the lungs is a key strategy to mitigate systemic toxicity and improve therapeutic efficacy. Cutting-edge technologies include nanoparticle-based targeted delivery, responsive smart delivery, and inhaled local delivery.

Progress has been made in nanoparticle technology for PF. Gold nanoparticles loaded with imatinib significantly enhance the anti-fibrotic efficacy of imatinib, inhibiting fibroblast and macrophage proliferation^253^. Studies have shown that intratracheal administration of pirfenidone nanoparticles results in significantly higher lung pirfenidone levels compared to pirfenidone solution. Nanoparticles maintain sustained delivery of pirfenidone in the lungs and enhance its anti-fibrotic effects^254^. Advances in nanoparticle targeting for LC have also been reported. A nanostructured lipid nanocarrier-based system (NLCS) has been proposed for efficient tumor-targeted local delivery of anti-tumor drugs and specific siRNA mixtures, specifically targeting lung cancer cells to effectively inhibit tumor growth and prevent adverse effects on healthy organs 255.

Additionally, progress has been made in responsive smart delivery systems. Wang et al. developed a gold nanoparticle-based smart responsive delivery system that precisely releases paclitaxel in the weakly acidic tumor microenvironment, reducing damage to normal tissues and side effects^256^. Li et al. developed a folic acid-modified ultrasound-responsive drug delivery system for delivering siRNA encoding signal regulatory protein alpha‌ (SIRPα) mRNA and immune adjuvant Fe₃O₄ nanoparticles^257^. Under ultrasound conditions, nanobubbles effectively transfect macrophages, inhibiting SIRPα mRNA and protein expression, promoting macrophage phagocytosis, and synergistically reversing M2 polarization to enhance immunotherapy in NSCLC.

Currently, inhaled local delivery methods such as dry powder inhalation and aerosol gels are less commonly used in PF and LC. However, given that inhaled administration delivers drugs directly to the lungs with rapid absorption, quick onset, no first-pass hepatic metabolism, and minimal side effects, future research could explore the role of inhaled local delivery systems in PF and LC.

Balancing anti-fibrotic and anti-tumor therapies requires overcoming the dual challenges of mechanism interaction and toxicity management, and organ-specific delivery systems provide revolutionary tools for this purpose. Future efforts should focus on precise sequential therapy, smart responsive carriers, and clinical translation standards to drive a paradigm shift from "conflicting therapies" to "synergistic eradication."

## 7. Conclusion​​

The interaction between the PFME and LC is a multidimensional, dynamically evolving vicious cycle. This review systematically elucidates its core mechanisms: the fibrotic microenvironment drives lung cancer initiation through chronic inflammation, genomic mutations, epigenetic reprogramming, mechanical signaling, immune suppression, and stem cell niches; conversely, tumor cells promote fibrosis progression by secreting pro-fibrotic factors, remodeling the ECM, and reprogramming metabolism. This bidirectional interaction not only explains the high prevalence and poor prognosis of their co-occurrence but also provides novel perspectives for targeted therapy.

Interdisciplinary research is critical to breaking through current therapeutic bottlenecks. Single-cell and spatial multi-omics technologies have revealed the cellular interaction networks within the microenvironment; organoid and organ-on-a-chip models have enabled dynamic simulation of pathological interfaces; and AI-driven multi-modal data integration is driving innovation in personalized treatment strategies. However, translational medicine still faces significant challenges: How to balance the conflicting effects of anti-fibrotic and anti-tumor therapies? How to achieve organ-selective drug delivery? Resolving these issues requires deep integration of bioengineering, computational science, and clinical medicine.

Looking to the future, precision medicine strategies should focus on the following directions, with deeper integration of mechanistic insights and technological innovations to address the complexity of fibrosis-lung cancer crosstalk:

First, real-time microenvironment monitoring and spatiotemporal dynamic intervention: Beyond developing multi-target mechanically responsive nanodrugs, the next step involves applying in vivo imaging techniques to dynamically track the fibrosis-tumor interface and determine optimal drug administration timing. For example, the best time to administer inhibitors is when both ECM stiffness and myofibroblast activation reach thresholds that promote tumor growth; continuous biopsies combined with scRNA-seq to observe temporal changes in cell states can dynamically adjust treatment regimens. If scRNA-seq reveals a surge in pro-inflammatory macrophages during anti-fibrotic therapy, combining it with colony-stimulating factor-1 receptor (CSF-1R) inhibitors to suppress this immunosuppressive subset can enhance efficacy.

Second, multi-omics-guided predictive stratified therapy: After molecular classification of "fibrosis-lung cancer" based on spatial transcriptomic and radiomic features, proteomics and metabolomics data can further refine treatment strategies for different subtypes. Specifically, tumors with a "sclerotic" microenvironment (characterized by LOXL2 expression and low hyaluronan levels) may be more sensitive to combined LOXL2 inhibition and hyaluronidase therapy, while "inflammatory" subtypes (with elevated IL-6 and TNF-α levels) may respond better to anti-fibrotic therapies.

Third, intelligent treatment systems with closed-loop optimization: A reinforcement learning model should be developed and trained to integrate multi-dimensional patient data—including clinical parameters, molecular features, and real-time physiological signals—to dynamically adjust combinations of anti-fibrotic and immunotherapy. For instance, if LOXL2 inhibition induces pneumonia, the model can intelligently recommend reducing anti-PD-1 dosage to balance efficacy and toxicity. These systems will also account for inter-patient variability; for example, a smoker with EGFR mutations may require a different immunotherapy backbone compared to a non-smoker, a decision guided by predictive models trained on large-scale multi-omics datasets.

Fourth, targeting the stem cell-niche axis to disrupt fibrosis-tumor crosstalk: ATII stem cells and their derived microenvironment are critical drivers of both fibrosis and cancer. Future interventions may include: 1 Niche disruption: Using antibody-drug conjugates targeting ATII cell surface markers (e.g., surfactant protein C) to selectively deplete cancer-prone stem cells while sparing normal progenitors; 2 Epigenetic reprogramming: Applying small-molecule inhibitors of histone methyltransferases or DNA methyltransferases to reverse abnormal gene expression caused by excessive ROS; 3 Metabolic reprogramming: Blocking glutamine metabolism in ATII-derived fibroblasts to starve the TME of energy required for both fibrosis progression and tumor growth.

Fifth, overcoming treatment resistance through combinatorial innovation: Beyond sequential anti-fibrotic and anti-tumor therapies, exploring synergistic combinations is key. Examples include: 1 Immunotherapy + anti-fibrosis: PD-1 inhibitors may fail in fibrotic tumors due to physical exclusion by dense ECM; co-administration with collagen-degrading enzymes or MMP activators can degrade ECM components (e.g., collagen), enhancing immune cell infiltration; 2 Targeted therapy + metabolic reprogramming: EGFR inhibitors (e.g., gefitinib) can be paired with PFKFB3 inhibitors to block aerobic glycolysis in both tumor cells and CAFs, depriving tumors of metabolic support; 3 Radiotherapy + fibrosis reversal: Hypo-fractionated radiotherapy can induce immunogenic cell death in tumor cells, while low-dose anti-TGF-β antibodies mitigate radiation-induced lung fibrosis.

Sixth, improving prognosis and quality of life through exercise: In addition to conventional treatments, reasonable exercise helps improve lung function. Exercise needs to be designed with individualized regimens. For example, for patients with poor physical performance status, exercise can start with low-intensity resistance training; patients with severe pulmonary fibrosis need to avoid high-intensity interval training to prevent hypoxemia exacerbation caused by hyperventilation; meanwhile, real-time monitoring technologies can be combined to dynamically adjust exercise regimens, ensuring that patients are not harmed. Additionally, the combined application of exercise with existing therapies (e.g., anti-fibrotic drugs,ICIs) can be further explored in the future. For instance, whether pre-exercise conditioning can enhance the tumor infiltration rate of PD-1 inhibitors, or whether the combination of exercise and MMP activators can accelerate ECM remodeling to improve drug delivery efficiency, providing a scientific basis for integrating "exercise prescriptions" into precision oncology management.

Finally, promoting clinical research progress using human-relevant models: To accelerate clinical translation, organoids and microphysiological systems should recapitulate the spatiotemporal dynamic evolution of fibrosis-lung cancer crosstalk. For example, "fibroblast-tumor organoids" co-cultured with patient-derived ECM and immune cells can simulate the transition from fibrosis to malignancy and test combinatorial therapies in a context-dependent manner.

In summary, unraveling the fibrosis-lung cancer vicious cycle demands a holistic approach that integrates real-time microenvironmental insights, multi-omics-driven personalization, intelligent therapeutic decision-making, and innovative niche-targeted strategies. Only through such deep mechanistic understanding and technological synergy can we transform the paradigm from managing co-morbid disease to achieving genuine "radical reversal"—restoring normal lung architecture, eliminating malignant clones, and preventing recurrence.

## Figures and Tables

**Table 1:** Incidence of class-specific adverse events associated with EGFR-TKIs for treatment of NSCLC.

EGFR-TKIs	n	Incidence of rash (%)	Incidence of Diarrhea (%)	AST/ALT Elevation (%)	Incidence of ILD (%)
Gefitinib	580	70.0	45.5	38.3	3.4
Erlotinib	276	92.4	51.1	36.6	4.0
Afatinib	160	88.0	91.0	10.0	1.3
Osimertinib	279	58.0	58.0	7.5	4.0

ILD: interstitial lung diseases. (Note. Modified from Ohmori et al.^162^ (2021) under the terms of the Creative Commons Attribution International License (CC BY 4.0).)

**Table 2:** Summary of Key Biomarkers

Biomarker​	Association Type ​	Associated Diseases ​	Main Functions/Mechanisms ​	Clinical Research	Reference
MMP-7​	Fibrosis-related	lung adenocarcinoma and NSCLC	Secreted proteolytic enzymes degrade ECM components; promote tumor cell invasion, metastasis, and angiogenesis	NSCLC tissues showed a positive expression rate of 68.89% (vs. 14% in normal lung tissues); the iMVD in the positive group was 46.2±6.77 (vs. 30.8±7.54 in the negative group, P<0.001)	(164-166)
KL-6​	Fibrosis-related	lung adenocarcinoma and TR-ILD	MUC1, expressed in ATII cells, regulates the stemness and paclitaxel resistance of lung adenocarcinoma through the MUC1-EGFR-NF-κB/MAPK signaling pathway	IPF combined with lung cancer patients exhibit poor prognosis with elevated KL-6; Nintedanib treatment stabilizes KL-6 levels at 24 months	(167-172)
CEA​	Tumor-related	LC and ILD	CEA family members, with elevated serum levels, are associated with both malignant and non-malignant diseases (e.g., PF)	Serum CEA levels show a downward trend in PF, lung cancer, and other patients; elevated levels are associated with both malignant and non-malignant diseases	(173-175)
CYFRA21-1​	Tumor-related	LC and IPF	Alveolar epithelial injury releases products that reflect epithelial damage and repair	Serum CYFRA21-1 levels in IPF patients are associated with disease progression/mortality and localized to proliferative epithelium	(176)
PD-L1​	Tumor-related	LC and IPF	Immune suppressor molecules, inhibit T cell activation; upregulated in IPF via EMT-induced PF; PD-1/PD-L1 inhibitor targets	IPF patients exhibit PD-L1 upregulation in fibrotic lung tissue and serum; the PD-L1-high (≥80%) subgroup in the M7824 trial achieved an ORR of 85.7%	(177-179)

**Table 3:** Prevalence of LC in IPF patients.

Study	Number of IPF patients	Number of Patients with LC-IPF	Prevalance of LC (%)
Nagai	99	31	31.3
Matsusitha	20	ND	48.2
Park	281	63	22.4
Le Jeune	1064	29	2.7
Ozawa	103	21	20.4
Lee	1685	70	6.8
Kreuter	265	42	16
Tomasetti	181	23	13
Yoon	1108	27	2.8
Kato	632	70	11.1

ND: not determined. (Note. From Ballester et al.^134^ (2019) under the terms of the Creative Commons Attribution International License (CC BY 4.0).)
